# ACE‐mediated Glycosylation Stabilizes PSAP To Promote GPR37‐dependent Macrophage‐Nucleus Pulposus Cells Crosstalk and TGFβ Signaling in Alleviating Intervertebral Disc Degeneration

**DOI:** 10.1002/advs.202510662

**Published:** 2025-10-14

**Authors:** Youfeng Guo, Feng Wang, Bijun Wang, Yu Zhou, Chao Wang, Tao Hu, Desheng Wu

**Affiliations:** ^1^ Department of spine surgery Shanghai East Hospital School of Medicine Tongji University Shanghai 200092 China; ^2^ Department of medical genetics School of Medicine Tongji University Shanghai 200092 China; ^3^ School of Materials Science and Engineering Shanghai University Shanghai 200444 China

**Keywords:** angiotensin‐converting enzyme, G protein‐coupled receptor 37, glycosylation, intervertebral disc degeneration, prosaposin

## Abstract

Intervertebral disc degeneration (IDD) represents a complex pathological process involving impaired cellular homeostasis and extracellular matrix dysregulation. This study elucidates a previously unrecognized regulatory axis wherein angiotensin‐converting enzyme (ACE) modulates prosaposin (PSAP) stability through coordinated post‐translational modifications. Mechanistically, ACE deficiency enhances *O*‐GlcNAc transferase (OGT)‐mediated glycosylation of PSAP at critical serine residues, which in turn suppresses E3 ubiquitin ligase Casitas B‐lineage lymphoma (CBL)‐dependent ubiquitination and proteasomal degradation. The stabilized PSAP protein engages G protein‐coupled receptor 37 (GPR37) on macrophages to promote anti‐inflammatory M2 polarization through ERK/SMAD2/3 signaling cascades, while concomitantly stimulating transforming growth factor‐β (TGFβ) secretion. This paracrine signaling establishes a reciprocal regulatory loop, as secreted TGFβ reinforces PSAP‐Sortilin mediated trafficking in nucleus pulposus cells via PI3K/AKT pathway activation. In vivo therapeutic intervention using engineered PSAP and GPR37 gene‐editing virus‐loaded hydrogels demonstrated significant improvements in disc structural integrity and matrix composition in preclinical IDD models, with these protective effects being dependent on GPR37 receptor activation. The findings reveal the ACE‐PSAP‐GPR37 axis as a fundamental regulatory circuit in disc homeostasis, providing new insights into the molecular pathogenesis of IDD while establishing a conceptual framework for targeted therapeutic development.

## Graphical abstract

ACE can modulate the CBL‐mediated K48 ubiquitination degradation of PSAP by altering its glycosylation levels in NP cells. As a result, NP cells secrete PSAP, which interacts with GPR37 on macrophage surfaces, facilitating their polarization toward the M2 phenotype. These M2 macrophages subsequently secrete TGFβ, which exerts feedback effects on the NP cells.

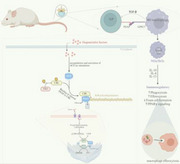

## 1 Background

Intervertebral disc degeneration (IDD) constitutes a principal factor contributing to spinal degenerative disorders, with its incidence rates increasing concomitantly with advancing age and the aging of the population.^[^ ^1^, ^2^ ^]^ Up to 80% of individuals experience low back pain at some point in their lifetime, a condition predominantly attributed to IDD.^[^ ^3^, ^4^ ^]^ This condition not only results in chronic pain and mobility limitations but may also lead to complications such as spinal stenosis, disc herniation, foraminal narrowing, or neural compression, which can significantly impair quality of life and work capacity. The economic burden is considerable, as conservative treatments merely provide temporary symptom relief without addressing the underlying disease, leading to prolonged reliance on healthcare services and placing significant strain on medical systems. More importantly, the pathogenesis of IDD is not yet fully elucidated. Although inflammation, oxidative stress, cellular senescence, and abnormal mechanical loading are recognized as contributing factors, the primary drivers and regulatory networks remain inadequately understood.^[^ ^5^ ^]^

Angiotensin‐converting enzyme (ACE) is a well‐known enzyme that plays a crucial role in the renin‐angiotensin system (RAS), which regulates blood pressure and fluid balance. ACE converts angiotensin I to the potent vasoconstrictor angiotensin II, thereby influencing cardiovascular homeostasis.^[^ ^6^ ^]^ In addition to its role in the RAS, ACE has been implicated in various physiological and pathological processes, including inflammation, immune regulation, and tissue repair.^[^ ^7^, ^8^, ^9^ ^]^ Our previous research data have shown that ACE knockdown in nucleus pulposus (NP) cells can improve the degenerative and senescent phenotypes of NP cells.^[^ ^10^ ^]^ In addition, ACE may influence the content of O‐GlcNAc transferase (OGT) protein and regulate the overall level of O‐GlcNAc modification in cells, which may indirectly affect the stability and activity of IDD‐related proteins.

Prosaposin (PSAP), a multifunctional glycoprotein encoded on chromosome 10q21‐q22, serves as the precursor for four saposin proteins (saposin A‐D).^[^ ^11^ ^]^ Within lysosomes, PSAP is processed to produce saposin A‐D fragments, which subsequently activate specific hydrolases. These hydrolases are crucial for the degradation of sphingolipids, thereby underscoring the pivotal role of PSAP in sphingolipid metabolism. PSAP is a multifunctional glycoprotein widely involved in cellular signaling and physiological regulation. One of its functional characteristics is the ability to regulate cell survival, proliferation, and differentiation through multiple signaling pathways.^[^ ^12^ ^]^ Research shows that PSAP and its derivatives can significantly activate the mitogen‐activated protein kinase (ERK) pathway.^[^ ^13^, ^14^ ^]^ For example, in various cell types, PSAP treatment promotes ERK1/2 phosphorylation, thereby enhancing cell survival and proliferation capacity.^[^ ^15^ ^]^ This effect has been observed in neuronal cells, tumor cells, and immune cells.^[^ ^16^, ^17^ ^]^ Furthermore, secreted PSAP interacts with receptors, including the G protein‐coupled receptor 37 (GPR37), to facilitate neurotrophic support, maintain myelin integrity, modulate inflammatory responses, regulate apoptotic processes, and promote tissue repair.^[^ ^18^ ^]^ GPR37, an orphan receptor broadly expressed in neural and immune systems,^[^ ^19^ ^]^ emerged as a focus when investigating NP cells‐macrophages crosstalk in intervertebral discs (IVDs). In macrophages, the activation of GPR37 facilitates the transition from the M1 to the M2 phenotype, augments phagocytic activity, diminishes the production of pro‐inflammatory cytokines, and increases the levels of anti‐inflammatory mediators.^[^ ^20^ ^]^ Moreover, GPR37's role in infection and sepsis has garnered widespread attention.^[^ ^21^ ^]^ Research indicates that GPR37 activation significantly enhances macrophage phagocytosis of plasmodium‐infected red blood cells, effectively alleviating malaria‐induced sepsis symptoms.^[^ ^22^ ^]^ In bacterial infection contexts, GPR37 also plays an important role by improving macrophage phagocytic efficiency and significantly reducing infection‐induced inflammatory responses.^[^ ^21^, ^22^ ^]^ PSAP is extensively distributed in the brain, liver, kidneys, and prostate, where it operates within the extracellular matrix (ECM), lysosomal, and cell membrane compartments.^[^ ^23^, ^24^, ^25^ ^]^ PSAP dysfunction is associated with various diseases: genetic mutations lead to lysosomal storage disorders like Gaucher disease and metachromatic leukodystrophy, and its role in macrophage regulation may contribute to inflammatory conditions like atherosclerosis.^[^ ^25^, ^26^ ^]^

Glycosylation is a common post‐translational modification that affects protein stability, folding, trafficking, and interactions with other proteins.^[^ ^27^ ^]^ Glycosylation modifications can reduce ubiquitination levels by altering protein conformation and decreasing binding affinity with E3 ubiquitin (Ub) ligases. For example, glycosylation may stabilize protein structure or enhance deubiquitinating enzymes binding affinity, thereby protecting proteins from degradation.^[^ ^28^, ^29^ ^]^ Additionally, glycosylation can reduce protein recognition and degradation by proteasomes by affecting proteasome function or modulating intracellular protein trafficking and localization.^[^ ^30^ ^]^ These mechanisms collectively enable glycosylated proteins to exist more stably within cells, playing important roles in various physiological and pathological processes.

Macrophages, which are essential components of the immune system, play a crucial role in the clearance of pathogens, the removal of cellular debris, and the regulation of inflammatory responses. In IDD, macrophage phenotypic polarization and cytokine secretion critically influence disease progression.^[^ ^31^ ^]^ Although healthy IVDs contain a minimal number of macrophages, degenerating NP tissue releases damage‐associated molecular patterns that facilitate their recruitment and activation.^[^ ^20^ ^]^ Macrophages undergo polarization into either the pro‐inflammatory M1 phenotype, characterized by the secretion of interleukin‐1β (IL‐1β), tumor necrosis factor‐α (TNF‐α), and matrix metalloproteinases (MMPs), which contribute to the exacerbation of ECM degradation and inflammation, or the anti‐inflammatory M2 phenotype, which is associated with the release of IL‐10 and transforming growth factor‐β (TGFβ), thereby facilitating tissue repair. Research indicates an imbalance between M1 and M2 macrophages in degenerated IVDs, where atypical interactions between macrophages and NP cells expedite cellular senescence and apoptosis.^[^ ^32^ ^]^ Therefore, targeting macrophage polarization or associated inflammatory pathways constitutes a promising therapeutic strategy for IDD. However, a more comprehensive understanding of the underlying mechanisms is necessary.

Given the established role of ACE in post‐translational modifications, we hypothesize that ACE deficiency stabilizes PSAP through glycosylation modifications, promoting its secretion from NP cells. The secreted PSAP then interacts with GPR37 receptors on macrophages to induce anti‐inflammatory polarization, thereby establishing a protective crosstalk between NP cells and macrophages that alleviates IDD. This study aims to elucidate the molecular mechanisms by which ACE regulates PSAP stability through glycosylation, investigate how PSAP‐GPR37 interaction mediates NP cells‐macrophages communication, and explore the therapeutic potential of targeting this pathway for disc degeneration treatment.

## 2 Results

### 2.1 Relationship between ACE, IDD, and PSAP

**Figure 1:**
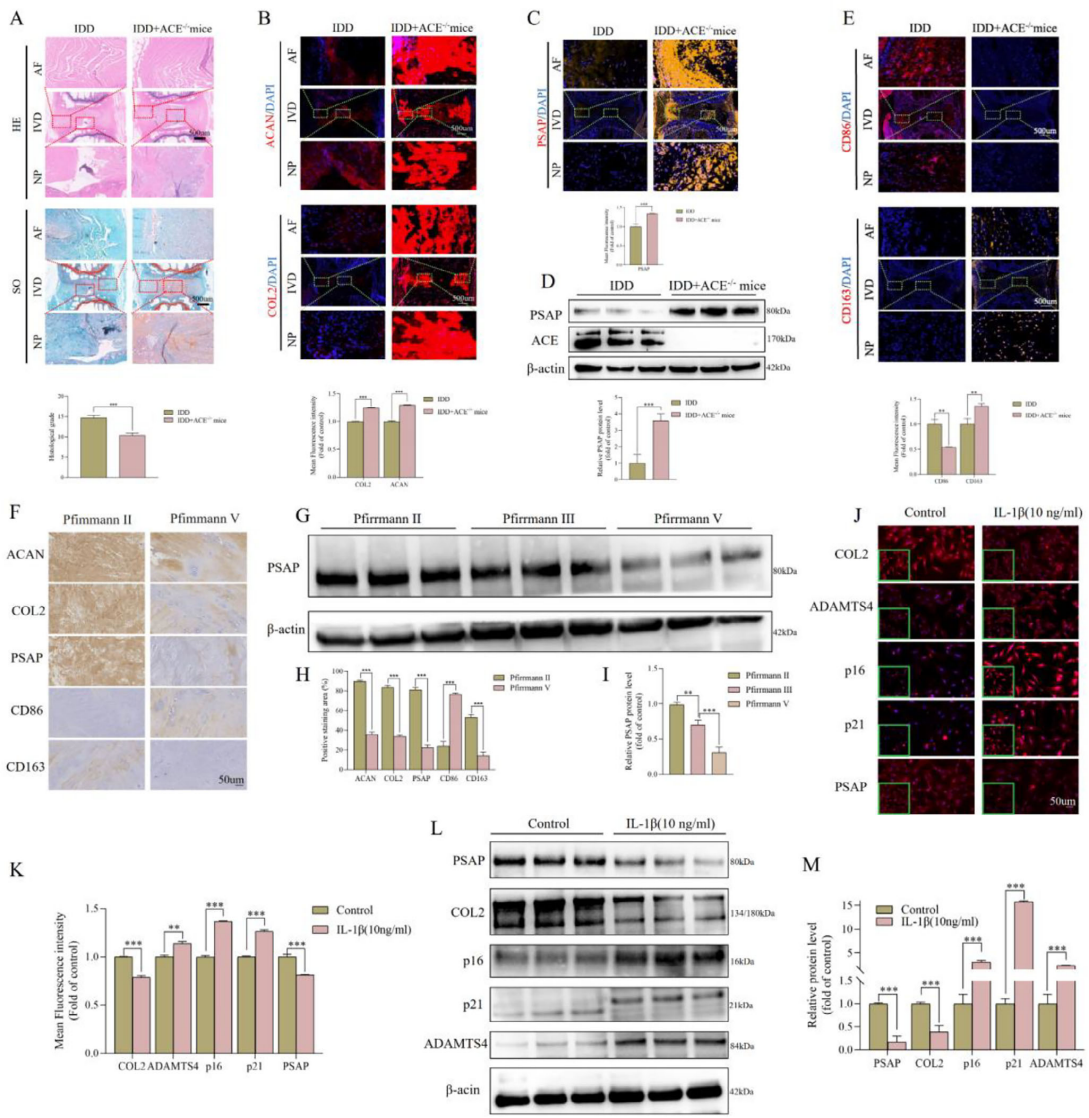
Alterations in PSAP expression levels and the degree of macrophage infiltration in ACE knockout mice, as well as in degenerated NP tissues and cells. A,B) Representative HE and SO staining images, as well as COL2 and ACAN tissue IF staining images and semi‐quantitative analysis of ACE knockout mice and wild‐type mice after needle puncture‐induced IDD modeling (n = 6). C) Representative PSAP tissue IF staining images and semi‐quantitative analysis of ACE knockout mice and wild‐type mice after needle puncture‐induced IDD modeling (n = 6). D) PSAP protein expression levels and band gray values in IVD of ACE knockout mice and wild‐type mice after needle puncture‐induced IDD modeling were semi‐quantitatively analyzed using ImageJ (n = 3). E) Representative macrophage infiltration tissue IF staining images and semi‐quantitative analysis of ACE knockout mice and wild‐type mice after needle puncture‐induced IDD modeling (n = 6). F and H) Representative IHC staining images and semi‐quantitative analysis of ECM (COL2 and ACAN) expression, PSAP expression and macrophage infiltration in human NP tissues section with different degeneration degrees (n = 3). G and I) From the harvested different degrees of degenerated human NP tissues, proteins were extracted and the expression level of PSAP protein was detected, and the band gray values were semi‐quantitatively analyzed using ImageJ (n = 3). J–M) In vitro analysis of PSAP and related (ADAMTS4 and COL2) and senescence markers (p16 and p21) levels by cellular IF staining and Wb after IL‐1β (10 ng mL^−1^) treatment of SD rat‐derived NP cells for 72 h. Fluorescence intensity and band gray values were semi‐quantitatively analyzed using ImageJ (n = 3). All data are expressed as the mean ± SD. Comparisons between two groups were made using the unpaired two‐tailed Student's *t*‐test, whereas comparisons among multiple groups were conducted using one‐way analysis of variance followed by Tukey's post hoc test. A *p*‐value of less than 0.05 was considered indicative of statistical significance. ^*^ *p* < 0.05, ^**^ *p* < 0.01, ^***^ *p* < 0.001, while “ns” indicates a lack of statistical significance. PSAP, Prosaposin; ACE, Angiotensin‐converting enzyme; NP, Nucleus pulposus; HE, Hematoxylin and eosin; SO, Safranine O‐Fast Green; COL2, Collagen type II; ACAN, Aggrecan; IF, Immunofluorescence; IVD, Intervertebral disc; IDD, Intervertebral disc degeneration; IHC, Immunohistochemistry; ECM, Extracellular matrix; ADAMTS4, A Disintegrin and Metalloproteinase with Thrombospondin Motifs 4; p16, Cyclin‐dependent kinase inhibitor 2A; p21, Cyclin‐dependent kinase inhibitor 1; Wb, Western blot; IL‐1β, Interleukin‐1β.

We effectively established an IDD model using needle puncture and validated its effectiveness through hematoxylin and eosin (HE) and safranin O/fast green (SO) staining to investigate the effect of ACE knockout on IDD (**Figure** **1**A,B). The quantitative assessment of Western blot (Wb) and tissue immunofluorescence (IF) revealed that IVD tissues from ACE knockout mice, within the context of a puncture‐induced IDD model, displayed significantly elevated levels of PSAP (Figure 1C,D). Additionally, there was a notable increase in the proportion of M2 macrophages (Figure 1E). These findings suggest that the modulation of macrophage polarization and PSAP levels through ACE inhibition could potentially alleviate the progression of IDD. The immunohistochemistry (IHC) of human NP tissues revealed a negative correlation between the severity of IDD and the expression of PSAP (Figure 1F,H). Specifically, more severe IDD was associated with reduced PSAP levels (Figure 1F,H), decreased M2 macrophages, and increased M1 macrophages. The Wb analysis confirmed that PSAP is progressively downregulated as IDD worsens in human NP tissues across various degeneration grades (Figure 1G,I). The expression of PSAP protein in rat‐derived NP cells was substantially reduced by IL‐1β treatment, as evidenced by in vitro experiments (Figure 1J–M). After modeling NP cells with TNF‐α (10 ng mL^−1^), there was a notable decrease in PSAP protein levels (Figure S1A, Supporting Information). These results highlight PSAP's crucial role in IDD pathogenesis and suggest that ACE knockout might alleviate IDD by influencing PSAP expression and macrophage polarization.

### 2.2 PSAP Functional Validation

**Figure 2:**
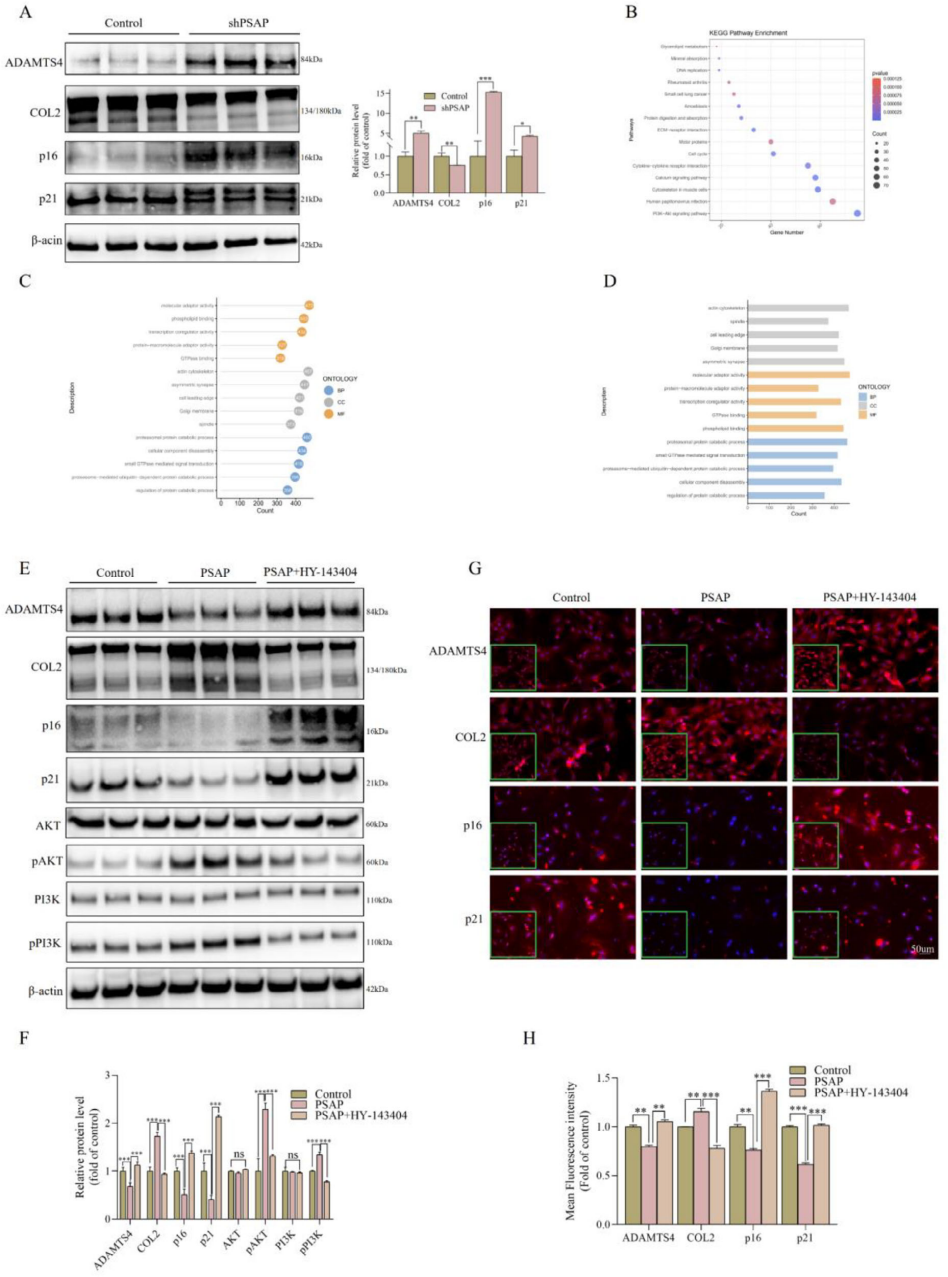
PSAP functional validation. A) Detection of degeneration (ADAMTS4 and COL2) and senescence (p16 and p21) marker levels in SD rat‐derived NP cells after shPSAP (lentivirus carrying PSAP shRNA) transfection for 72 h. Band gray values were semi‐quantitatively analyzed using ImageJ (n = 3). B–D) We performed transcriptome sequencing comparison between PSAP recombinant protein (10 µm)‐treated SD rat‐derived NP cells and control groups, followed by KEGG (B) and GO (C and D) analyses of the differentially expressed genes. For KEGG pathway analysis, it is first necessary to map the differentially expressed genes from both the control and PSAP recombinant protein (10 µm) groups to pathway entries in the KEGG database, calculate the enrichment *p*‐value for each pathway using hypergeometric tests or Fisher's exact tests, and obtain the *q*‐value after multiple testing correction. In the generated KEGG bubble plot, the x‐axis typically represents the number of differentially expressed genes in that pathway, the y‐axis displays specific pathway names, the bubble size represents the number of differentially expressed genes in that pathway, and the color intensity corresponds to ‐log10(q‐value) or ‐log10(*p*‐value), with darker colors indicating greater statistical significance. For GO analysis, differentially expressed genes are systematically classified into three main ontologies: molecular function, cellular component, and biological process. The display format of GO bubble plots is similar to that of KEGG, with the x‐axis representing the number of differentially expressed genes, the y‐axis showing specific GO terms, the bubble size indicating the number of differentially expressed genes included in that term, and the color intensity representing the enrichment significance. E–H) After PSAP recombinant protein (10 µm) treatment of SD rat‐derived NP cells for 48 h, PI3K inhibitor HY‐143404 (10 µm) was added for treatment. Wb and IF staining were used to analyze the corresponding degeneration (ADAMTS4 and COL2) and senescence (p16 and p21) marker levels, and ImageJ was used for semi‐quantitative analysis (n = 3). All data are expressed as the mean ± SD. Comparisons between two groups were made using the unpaired two‐tailed Student's *t*‐test, whereas comparisons among multiple groups were conducted using one‐way analysis of variance followed by Tukey's post hoc test. A *p*‐value of less than 0.05 was considered indicative of statistical significance. ^*^ *p* < 0.05, ^**^ *p* < 0.01, ^***^ *p* < 0.001, while “ns” indicates a lack of statistical significance. PSAP, Prosaposin; ADAMTS4, A Disintegrin and Metalloproteinase with Thrombospondin Motifs 4; COL2, Collagen Type II; p16, Cyclin‐Dependent Kinase Inhibitor 2A; p21, Cyclin‐Dependent Kinase Inhibitor 1; NP, Nucleus Pulposus; KEGG, Kyoto Encyclopedia of Genes and Genomes; GO, Gene Ontology; PI3K, Phosphoinositide 3‐Kinase; Wb, Western blot; IF, Immunofluorescence.

We successfully generated PSAP‐stably‐knockout rat‐derived NP cell lines through lentiviral transfection in order to investigate the regulatory function of PSAP in IDD. Wb and quantitative analysis showed that PSAP knockout worsened NP cells degeneration and significantly raised cellular senescence markers (**Figure** **2**A). It is important to note that the expression levels of senescence‐related proteins cyclin‐dependent kinase inhibitor 1 (p21) and cyclin‐dependent kinase inhibitor 2A (p16) were substantially upregulated in the PSAP knockout group compared to the control group, as demonstrated by Wb analysis. We performed transcriptome sequencing comparison between PSAP recombinant protein‐treated rat‐derived NP cells and control groups, followed by Kyoto Encyclopedia of Genes and Genomes (KEGG)and Gene Ontology (GO) analyses of the differentially expressed genes. KEGG pathway enrichment analysis (Figure 2B) revealed the enrichment of differentially expressed genes in multiple biological pathways, including the phosphatidylinositol 3‐kinase/protein kinase B (PI3K‐AKT) signaling pathway, cytoskeleton in muscle cells, calcium signaling pathway, cytokine–cytokine receptor interaction, and cell cycle. The significance of pathways was measured by *p*‐values, with lower *p*‐values (such as those for the PI3K/AKT signaling pathway and cell cycle) indicating higher enrichment levels of these pathways in the gene set, suggesting their potentially important functional or regulatory roles in the biological context. GO analysis (Figure 2C,D) demonstrated the enrichment of differentially expressed genes related to protein degradation in biological processes, cellular components, and molecular functions. Significantly enriched terms included “protein catabolic process” and “proteasome‐mediated Ub‐dependent protein catabolic process,” indicating their importance in intracellular activities. The involved cellular components included the Golgi membrane, spindle, and actin cytoskeleton, suggesting these genes may function in these cellular structures. Regarding molecular functions, terms such as phospholipid binding, GTPase binding, transcription coregulator activity, and protein‐macromolecule adaptor activity emerged, demonstrating their diverse functions, including signal transduction, transcriptional regulation, and interactions with other macromolecules. In order to verify this discovery, we conducted a systematic comparison of three cell groups using Wb and IF experiments: the control group, PSAP group, and PSAP combined with PI3K inhibitor HY‐143404 treatment group. The experimental results demonstrated that PSAP significantly enhanced the phosphorylation levels of key proteins in the PI3K/AKT pathway, while the PI3K inhibitor HY‐143404 could partially counteract or reverse the protective effects of PSAP on NP cells (Figure 2E–H). Additionally, we validated the functional effects of the PI3K inhibitor HY‐143404, and corresponding Wb analysis demonstrated that the addition of HY‐143404 exacerbated the degeneration and senescence of NP cells (Figure S1B, Supporting Information). Moreover, combined treatment with PSAP and a PI3K inhibitor exacerbated senescence and degenerative phenotypes in NP cells to a greater extent than the PI3K inhibitor alone. This indicates that, beyond the effect of PI3K inhibition, PSAP itself exerts a protective function on NP cells that is partially abrogated when the PI3K pathway is blocked. These findings lend further support to the notion that PSAP acts, at least in part, via the PI3K pathway. The results of this study conclusively demonstrate that PSAP primarily regulates the activity of the PI3K/AKT signaling pathway, which in turn influences the degenerative process and senescence state of NP cells.

### 2.3 The Regulation of PSAP Glycosylation and Functional Properties by ACE Knockout

**Figure 3:**
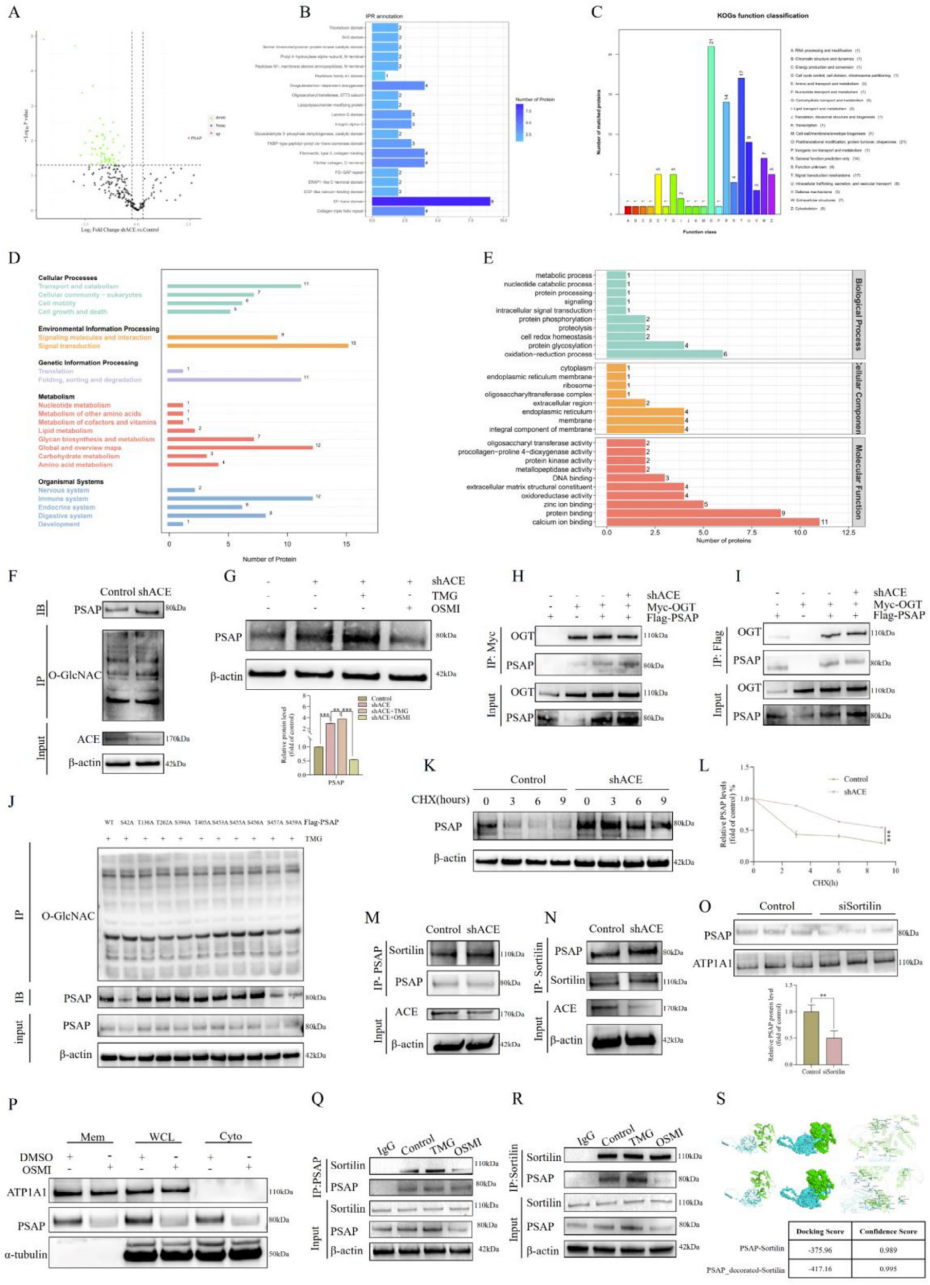
ACE‐mediated glycosylation modification and functional regulation mechanism of PSAP. We conducted a glycoproteomic sequencing screening of differentially glycosylated proteins comparison between shACE (lentivirus carrying ACE shRNA)‐treated SD rat‐derived NP cells and control groups and subsequent functional annotation. A) The volcano plot of glycoprotein differential analysis. In the volcano plot, the x‐axis represents the fold change (log2 value) of differentially expressed proteins, the y‐axis represents the *P*‐value (‐log10 value), with black dots indicating non‐significant proteins, red dots indicating upregulated proteins, and green dots indicating downregulated proteins. B) Diagram of IPR function annotation analysis. To provide more comprehensive domain annotation, Interproscan incorporates several widely used domain databases, using pattern structures or features to annotate domains of functionally unknown proteins. In the IPR plot, the x‐axis represents the number of proteins, and the y‐axis represents the annotated IPR entries. C) Diagram of KOG function annotation analysis. Through alignment in KOGs database, a protein sequence can be annotated to a specific cluster, with each cluster consisting of orthologous sequences, thereby allowing inference of the sequence's function. In the KOG plot, the x‐axis represents the functional categories of annotation, and the y‐axis represents the number of proteins annotated to the corresponding functions. D) Diagram of KEGG function annotation analysis. In the KEGG plot, the x‐axis represents the number of proteins, and the y‐axis represents the annotated KEGG entries (the primary biochemical metabolic pathways and signal transduction pathways). E) Diagram of GO function annotation analysis. In the GO plot, the x‐axis represents the number of proteins, and the y‐axis represents the annotated GO entries (cellular component, molecular function, and biological process). F) Detection of PSAP glycosylation levels after shACE transfection of SD rat‐derived NP cells in vitro. G) After shACE transfection of SD rat‐derived NP cells for 72 h, glycosylation agonist TMG (10 µm) or glycosylation inhibitor OSMI (25 mM) was used for treatment, followed by detection of PSAP protein levels. Band gray values were semi‐quantitatively analyzed using ImageJ (n = 3). H,I) IB analysis of cell lysates and anti‐Flag or anti‐Myc (as IP) derived from SD rat‐derived NP cells treated with shACE, Myc‐tagged OGT overexpression plasmid (Myc‐OGT), and/or Flag‐PSAP (Flag‐tagged PSAP overexpression plasmid). All input lysates were subjected to IB for OGT and PSAP. J) SD rat‐derived NP cells were co‐treated with TMG (10 µm) and Flag‐PSAP WT or its mutants, and cell lysates were subjected to IP and IB with the indicated antibodies. K,L) SD rat‐derived NP cells transfected with shACE were treated with CHX (50 µg mL^−1^) for specified durations, followed by IB of cell lysates to determine the relative levels of PSAP compared to β‐actin (n = 3). In the quantitative line graph, the x‐axis represents the CHX (50 µg mL^−1^) treatment time, while the y‐axis indicates the relative expression level of the target protein normalized to β‐actin (with the 0‐h value set as 100%). M,N) IB analysis of cell lysates and anti‐PSAP or anti‐Sortilin (as IP) derived from SD rat‐derived NP cells treated as indicated. All input lysates were subjected to IB for Sortilin and PSAP. O) After siSortilin transfection of SD rat‐derived NP cells in vitro, PSAP protein levels were detected and semi‐quantitatively analyzed using ImageJ (n = 3). P) Extract different cellular fractions (Mem, WCL, and Cyto) to analyze PSAP protein levels in SD rat‐derived NP cells, with or without OSMI treatment (25 mM). Q,R) IB analysis of cell lysates and anti‐PSAP or anti‐Sortilin (as IP) derived from SD rat‐derived NP cells treated as indicated. All input lysates were subjected to IB for Sortilin and PSAP. S) Molecular docking of dynamic modification omics was used to detect the binding free energy between glycosylated PSAP and Sortilin. The green structure represents the wild‐type PSAP protein, while the blue structure corresponds to the sortilin protein. All data are expressed as the mean ± SD. Comparisons between two groups were made using the unpaired two‐tailed Student's *t*‐test, whereas comparisons among multiple groups were conducted using one‐way analysis of variance followed by Tukey's post hoc test. A *p*‐value of less than 0.05 was considered indicative of statistical significance. ^*^ *p* < 0.05, ^**^ *p* < 0.01, ^***^ *p* < 0.001, while “ns” indicates a lack of statistical significance. ACE, Angiotensin‐converting enzyme; PSAP, Prosaposin; NP, Nucleus pulposus; TMG, Thiamet‐G; OSMI, *O*‐Glycosyltransferase inhibitor; IB, Immunoblotting; IP, Immunoprecipitation; OGT, O‐linked N‐acetylglucosamine transferase; CHX, Cycloheximide; Mem, membrane fraction; WCL, whole cell lysate; Cyto, cytoplasmic fraction; IPR, InterPro protein domain annotation; KOG, EuKaryotic orthologous groups.

We initially conducted a glycoproteomic sequencing screening of differentially glycosylated proteins comparison between shACE‐treated rat‐derived NP cells and normal control groups, in accordance with our previous discoveries that ACE inhibition induces protein glycosylation in NP cells.^[^ ^10^ ^]^ The results revealed that among all the proteins identified, only PSAP exhibited a significant elevation in glycosylation levels, as illustrated in **Figure** **3**A. The interpro protein domain annotation (IPR) annotation analysis revealed significant enrichment of EF‐hand domains and ECM‐interacting domains (such as fibrinogen‐like domains) among the differentially expressed glycoproteins, suggesting potential alterations in ECM composition or function (Figure 3B). Eukaryotic Orthologous Groups (KOG) annotation categorizes proteins into different functional groups, aiding in understanding the biological roles and mechanisms of glycoproteins in cells. KOG functional annotation analysis indicated that the differentially expressed proteins were primarily associated with post‐translational modification, protein turnover, chaperones, and intracellular signaling networks (Figure 3C). KEGG pathway analysis further confirmed that the differentially expressed proteins were significantly enriched in glycan biosynthesis and metabolism (critical for glycosylation modifications), protein folding, sorting, and degradation processes, and may influence intracellular signaling networks (Figure 3D). Additionally, many of these differentially expressed proteins play roles in the immune, digestive, and endocrine systems. GO analysis demonstrated that the differentially expressed proteins were enriched in protein degradation‐related terms across biological processes, cellular components, and molecular functions (Figure 3E). Specifically, at the biological process level, significantly enriched terms included protein glycosylation and redox processes, highlighting their critical roles in cellular activities. At the cellular component level, enriched terms involved the endoplasmic reticulum and membrane structures, suggesting that these proteins may function in these organelles or structures. At the molecular function level, enriched terms included zinc ion binding, protein binding, and calcium ion binding, indicating that the differentially expressed proteins may participate in ion binding, signal transduction, and interactions with other biomacromolecules. Experimental validation confirmed that PSAP glycosylation levels were significantly elevated in NP cells following shACE transfection (Figure 3F), which was consistent with the results shown in Figure 3A. Importantly, our observations revealed a positive correlation between PSAP protein expression and glycosylation levels within the ACE knockdown model, particularly when overall glycosylation levels were altered using glycosylation modulators such as Thiamet‐G (TMG) or the O‐Glycosyltransferase inhibitor (OSMI) (Figure 3G). Mechanistic studies have demonstrated that the knockdown of ACE significantly enhances the binding affinity between OGT and PSAP, as illustrated in Figure 3H,I. Through the integration of glycoproteomic data and cellular experiments, we successfully identified three crucial glycosylation sites on PSAP: Ser42, Ser457, and Ser459 (Figure 3J). In order to clarify the regulatory impact of ACE on PSAP stability, we conducted cycloheximide (CHX) chase experiments. The results of these experiments indicated that the removal of ACE significantly postponed the degradation of PSAP, as illustrated in Figure 3K,L. We conducted an analysis of transcriptome data following ACE knockdown and discovered that Sortilin expression was substantially upregulated (Figure S1D, Supporting Information), in accordance with our confirmation of their interaction (Figure S1C, Supporting Information) and previous reports on the significant role of Sortilin in PSAP secretion and transport.^[^ ^33^, ^34^, ^35^ ^]^ Sortilin and PSAP exhibited improved interaction in subsequent experiments (Figure 3M,N). Functional experiments confirmed that the localization of PSAP on the cell membrane was markedly reduced due to the knockout of Sortilin (Figure 3O). Further mechanistic studies revealed that the attachment of PSAP to the cell membrane was not only enhanced but also that its interaction with Sortilin was facilitated by an increase in overall cellular glycosylation levels (Figures 3P–R). This conclusion was substantiated by dynamic modification‐based molecular docking experiments, which revealed that post‐glycosylation, PSAP exhibited a significantly reduced binding free energy with Sortilin, indicating an enhanced binding affinity (Figure 3S). The molecular mechanism by which ACE influences the stability and transport of PSAP through glycosylation modification is systematically revealed by these results.

### 2.4 The Stability of the PSAP Protein is Regulated by Glycosylation Modifications

**Figure 4:**
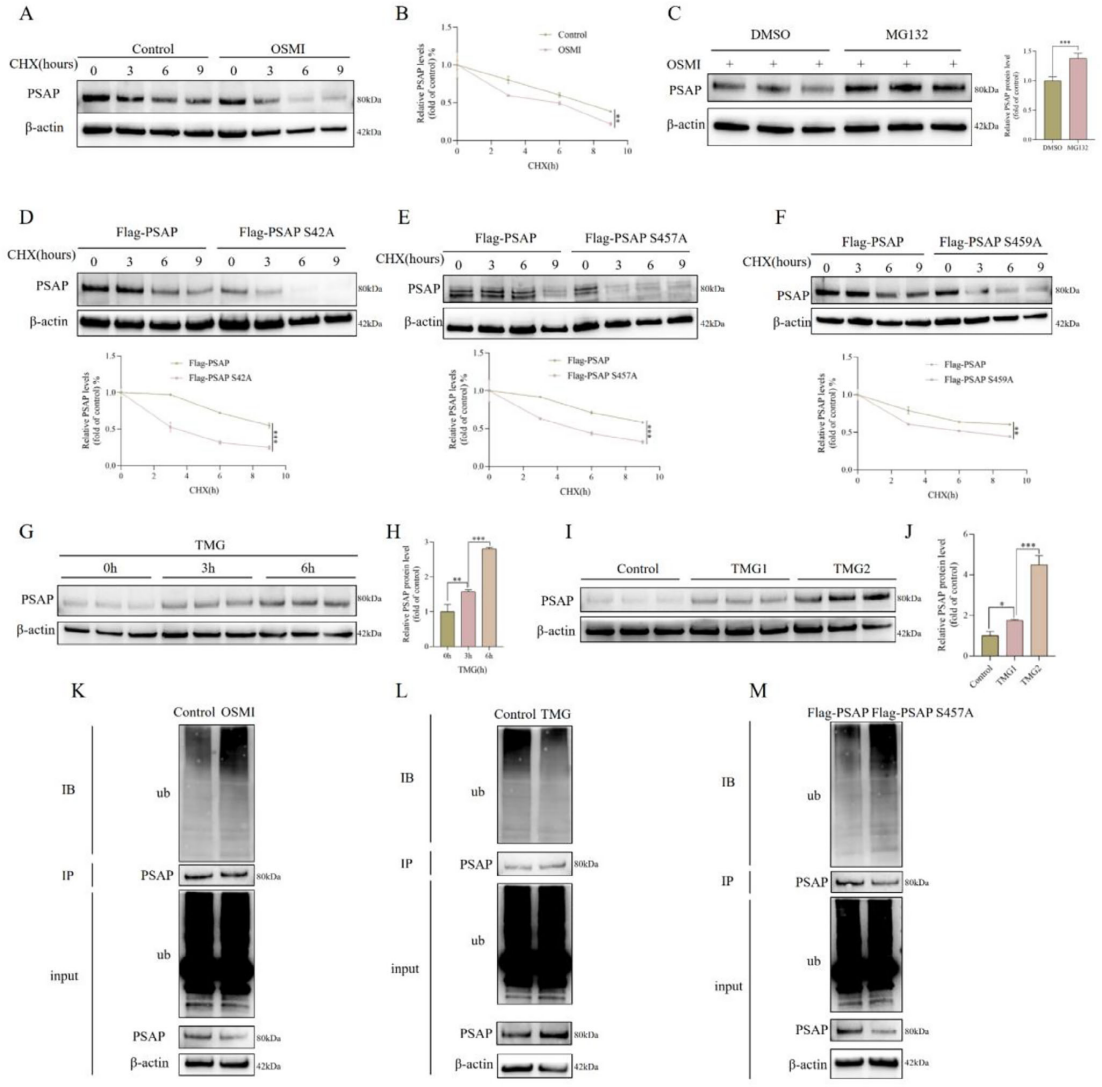
The effect of PSAP glycosylation levels on its own protein expression. A,B) SD rat‐derived NP cells treated with OSMI (25 mM) were treated with CHX (50 µg mL^−1^) for specified durations, followed by IB of cell lysates to determine the relative levels of PSAP compared to β‐actin (n = 3). In the quantitative line graph (B), the x‐axis represents the CHX (50 µg mL^−1^) treatment time, while the y‐axis indicates the relative expression level of the target protein normalized to β‐actin (with the 0‐h value set as 100%). C) SD rat‐derived NP cells were treated with OSMI (25 mM) for 48 h, followed by treatment with MG 132 (20 µm) for 6 h. Band gray values were semi‐quantitatively analyzed using ImageJ (n = 3). D) SD rat‐derived NP cells transfected with Flag‐tagged PSAP overexpression plasmid (Flag‐PSAP) or Flag‐tagged PSAP overexpression mutant plasmid (Flag‐PSAP S42A) were treated with CHX (50 µg mL^−1^) for specified durations, followed by IB of cell lysates to determine the relative levels of PSAP compared to β‐actin (n = 3). In the quantitative line graph, the x‐axis represents the CHX (50 µg mL^−1^) treatment time, while the y‐axis indicates the relative expression level of the target protein normalized to β‐actin (with the 0‐h value set as 100%). E) SD rat‐derived NP cells transfected with Flag‐PSAP or Flag‐PSAP S457A were treated with CHX (50 µg mL^−1^) for specified durations, followed by IB of cell lysates to determine the relative levels of PSAP compared to β‐actin (n = 3). In the quantitative line graph, the x‐axis represents the CHX (50 µg mL^−1^) treatment time, while the y‐axis indicates the relative expression level of the target protein normalized to β‐actin (with the 0‐h value set as 100%). F) SD rat‐derived NP cells transfected with Flag‐PSAP or Flag‐PSAP S459A were treated with CHX (50 µg mL^−1^) for specified durations, followed by IB of cell lysates to determine the relative levels of PSAP compared to β‐actin (n = 3). In the quantitative line graph, the x‐axis represents the CHX (50 µg mL^−1^) treatment time, while the y‐axis indicates the relative expression level of the target protein normalized to β‐actin (with the 0‐h value set as 100%). G,H) SD rat‐derived NP cells were treated with TMG (10 µm) for specified durations, followed by detection of PSAP protein levels (n = 3). I,J) After treatment of SD rat‐derived NP cells with different concentrations of TMG (concentration #1‐10 µm or concentration #2–15 µm), PSAP protein levels were detected (n = 3). K) SD rat‐derived NP cells underwent treatment with OSMI (25 mM) for a duration of 48 h. The cell lysate underwent IP using PSAP antibody, then followed by IB using Ub antibody. L) SD rat‐derived NP cells underwent treatment with TMG (10 µm) for a duration of 48 h. The cell lysate underwent IP using PSAP antibody, then followed by IB using Ub antibody. M) SD rat‐derived NP cells underwent transfection with Flag‐PSAP or Flag‐PSAP S457A for a duration of 72 h. The cell lysate underwent IP using PSAP antibody, then followed by IB using Ub antibody. All data are expressed as the mean ± SD. Comparisons between two groups were made using the unpaired two‐tailed Student's *t*‐test, whereas comparisons among multiple groups were conducted using one‐way analysis of variance followed by Tukey's post hoc test. A *p*‐value of less than 0.05 was considered indicative of statistical significance. ^*^ *p* < 0.05, ^**^ *p* < 0.01, ^***^ *p* < 0.001, while “ns” indicates a lack of statistical significance. NP, Nucleus pulposus; OSMI, *O*‐Glycosyltransferase inhibitor; CHX, Cycloheximide; IB, Immunoblotting; PSAP, Prosaposin; MG 132, Carbobenzoxy‐Leu‐Leu‐leucinal; TMG, Thiamet‐G; IP, Immunoprecipitation; Ub, Ubiquitin.

To assess the impact of PSAP glycosylation status on its stability, we designed and conducted a series of CHX chase experiments. At the outset, we observed that treatment with the glycosylation inhibitor OSMI not only markedly accelerated the degradation of PSAP protein but also substantially decreased its abundance, as illustrated in **Figure** **4**A,B. It is important to note that the proteasome inhibitor Carbobenzoxy‐Leu‐Leu‐leucinal (MG132) was able to effectively reverse the degradation of PSAP induced by OSMI, indicating that the Ub‐proteasome pathway may modulate PSAP stability through glycosylation (Figure 4C). Furthermore, point mutations were introduced at the three identified glycosylation sites of PSAP (Ser42, Ser457, and Ser459). The resulting mutants demonstrated enhanced rates of protein degradation, underscoring the critical role of these glycosylation sites in maintaining PSAP stability (Figure 4D–F). In accordance with this, the concentration and time‐dependent upregulation of PSAP protein levels were facilitated by the glycosylation activator TMG (Figure 4G–J). To elucidate the molecular mechanism by which glycosylation affects the stability of PSAP, we focused on examining the impact of overall cellular glycosylation levels on the ubiquitination status of PSAP. The experimental results indicated that TMG treatment substantially reduced endogenous PSAP ubiquitination, whereas OSMI treatment had the opposite effect (Figure 4K,L). The ubiquitination of endogenous PSAP was notably enhanced due to mutations at any glycosylation site of PSAP (Figure 4M). These results collectively verify that PSAP glycosylation preserves protein stability by preventing Ub‐mediated degradation.

### 2.5 Validation of PSAP Ubiquitination Types and Sites

**Figure 5:**
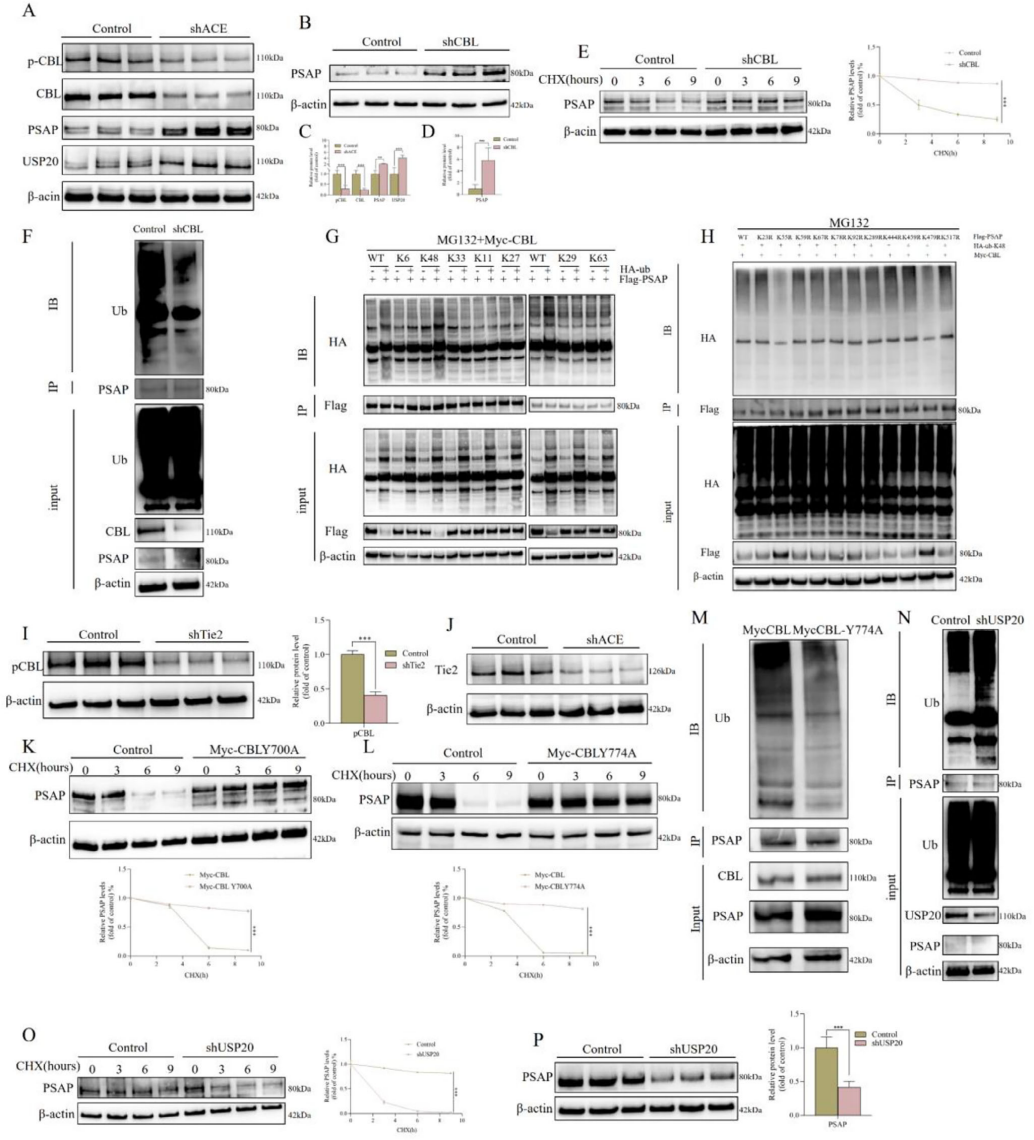
PSAP ubiquitination degradation mechanism. A,C) After shACE (lentivirus carrying ACE shRNA) transfection of SD rat‐derived NP cells in vitro, total CBL protein and its phosphorylated protein, PSAP and USP20 protein levels were detected. Band gray values were semi‐quantitatively analyzed using ImageJ (n = 3). B,D) After shCBL (lentivirus carrying CBL shRNA) transfection of SD rat‐derived NP cells in vitro, PSAP protein levels were detected (n = 3). E) SD rat‐derived NP cells transfected with shCBL were treated with CHX (50 µg mL^−1^) for specified durations, followed by IB of cell lysates to determine the relative levels of PSAP compared to β‐actin (n = 3). In the quantitative line graph, the x‐axis represents the CHX (50 µg mL^−1^) treatment time, while the y‐axis indicates the relative expression level of the target protein normalized to β‐actin (with the 0‐h value set as 100%). F) SD rat‐derived NP cells underwent transfection with shCBL for a duration of 72 h. The cell lysate underwent IP using PSAP antibody, then followed by IB using Ub antibody. G) SD rat‐derived NP cells were transfected with Myc‐tagged CBL overexpression plasmid (Myc‐CBL) and specified Ub mutants together with Flag‐tagged PSAP overexpression plasmid (Flag‐PSAP), then treated with MG 132 (20 µm) for 6 h. Cell lysates were subjected to IP and IB with specified antibodies. H) SD rat‐derived NP cells were transfected with Myc‐CBL and HA‐Ub‐K48 together with Flag‐PSAP WT or its mutants, then treated with MG 132 (20 µm) for 6 h. Cell lysates were subjected to IP and IB with the indicated antibodies. I) After shTie2 (lentivirus carrying Tie2 shRNA) transfection of SD rat‐derived NP cells in vitro, phosphorylation levels of CBL were detected. Band gray values were semi‐quantitatively analyzed using ImageJ (n = 3). J) After shACE transfection of SD rat‐derived NP cells in vitro, Tie2 protein levels were detected. K) SD rat‐derived NP cells transfected with Myc‐CBL or Myc‐tagged CBL overexpression mutant plasmid (Myc‐CBL Y700A) were treated with CHX (50 µg mL^−1^) for specified durations, followed by IB of cell lysates to determine the relative levels of PSAP compared to β‐actin (n = 3). In the quantitative line graph, the x‐axis represents the CHX (50 µg mL^−1^) treatment time, while the y‐axis indicates the relative expression level of the target protein normalized to β‐actin (with the 0‐h value set as 100%). L) SD rat‐derived NP cells transfected with Myc‐CBL or Myc‐CBL Y774A were treated with CHX (50 µg mL^−1^) for specified durations, followed by IB of cell lysates to determine the relative levels of PSAP compared to β‐actin (n = 3). In the quantitative line graph, the x‐axis represents the CHX (50 µg mL^−1^) treatment time, while the y‐axis indicates the relative expression level of the target protein normalized to β‐actin (with the 0‐h value set as 100%). M) SD rat‐derived NP cells underwent transfection with Myc‐CBL or Myc‐CBL Y774A for a duration of 72 h. The cell lysate underwent IP using PSAP antibody, then followed by IB using Ub antibody. N) SD rat‐derived NP cells underwent transfection with shUSP20 (lentivirus carrying USP20 shRNA) for a duration of 72 h. The cell lysate underwent IP using PSAP antibody, then followed by IB using Ub antibody. O) SD rat‐derived NP cells transfected with shUSP20 were treated with CHX (50 µg mL^−1^) for specified durations, followed by IB of cell lysates to determine the relative levels of PSAP compared to β‐actin (n = 3). In the quantitative line graph, the x‐axis represents the CHX (50 µg mL^−1^) treatment time, while the y‐axis indicates the relative expression level of the target protein normalized to β‐actin (with the 0‐h value set as 100%). P) After shUSP20 transfection of SD rat‐derived NP cells in vitro, PSAP protein levels were detected (n = 3). All data are expressed as the mean ± SD. Comparisons between two groups were made using the unpaired two‐tailed Student's *t*‐test, whereas comparisons among multiple groups were conducted using one‐way analysis of variance followed by Tukey's post hoc test. A *p*‐value of less than 0.05 was considered indicative of statistical significance. ^*^ *p* < 0.05, ^**^ *p* < 0.01, ^***^ *p* < 0.001, while “ns” indicates a lack of statistical significance. PSAP, Prosaposin; NP, Nucleus pulposus; CBL, Casitas B‐lineage lymphoma; USP20, Ubiquitin‐Specific‐Processing Protease 20; CHX, Cycloheximide; IB, Immunoblotting; IP, Immunoprecipitation; Ub, Ubiquitin; MG 132, Carbobenzoxy‐Leu‐Leu‐leucinal; Tie2, TEK receptor tyrosine kinase.

First, we identified the E3 Ub ligase Casitas B‐lineage lymphoma (CBL) as a specific binding partner of PSAP through protein interaction screening in order to further dissect the regulatory network of PSAP protein stability (Figure S1E, Supporting Information). It is important to note that the total protein level of CBL was substantially reduced in ACE‐knockdown rat‐derived NP cells, and its phosphorylation was also markedly downregulated (**Figures** **5**A,C and S1F,G, Supporting Information). This phenomenon was in stark contrast to the IL‐1β‐induced rat‐derived NP cells degeneration model, in which CBL expression was substantially upregulated (Figure S1H, Supporting Information). This indicates that CBL might play a role in the degradation of PSAP under pathological conditions, as illustrated in Figure S1H (Supporting Information). Through systematic functional validation experiments, we found that the knockout of CBL significantly elevated the levels of PSAP protein and prolonged its degradation rate. This finding was further corroborated by CHX chase experiments (Figure 5B,D,E). More significantly, the lack of CBL led to a pronounced reduction in the ubiquitination of PSAP, as illustrated in Figure 5F. Conversely, the Myc‐CBL WT transfection in conjunction with MG132 treatment plainly demonstrated that CBL regulates PSAP degradation through the proteasome pathway (Figure S1I, Supporting Information). CBL's critical function as a critical E3 Ub ligase in the regulation of PSAP protein homeostasis is collectively established by these findings.

Through a series of meticulously designed experiments, we successfully elucidated the intricate regulatory network governing PSAP stability at the molecular level. Initially, we verified that PSAP is predominantly ubiquitinated via K48 (Figure 5G) using a Ub mutant system. Subsequently, site‐directed mutagenesis screening enabled the identification of two pivotal ubiquitination sites, K55 and K479, as illustrated in Figure 5H. It is noteworthy that the interaction between CBL and PSAP was significantly attenuated by ACE knockdown (refer to Figure S1J, Supporting Information) as well as by the TMG‐induced increase in glycosylation levels (refer to). Conversely, the glycosylation inhibitor OSMI had the opposite effect (Figure S1L, Supporting Information). This phenomenon was further substantiated through PSAP glycosylation site mutation experiments (Figures S1P–R, Supporting Information), which unequivocally demonstrated that glycosylation regulates PSAP stability by modulating protein–protein interactions.

Research indicates that the phosphorylation of CBL family proteins is essential for substrate recognition, implying that their phosphorylation status plays a critical role in enzymatic activity.^[^ ^36^, ^37^ ^]^ To assess the impact of phosphorylation on CBL activity, we employed λ‐phosphatase to dephosphorylate immunoprecipitated CBL and PSAP. This treatment resulted in a marked reduction in their interaction, as illustrated in Figure S1M (Supporting Information). This motivated us to examine potential kinases that are accountable for the interaction between CBL and PSAP. In order to ascertain whether TEK receptor tyrosine kinase (Tie2) influences CBL, we monitored the phosphorylation levels of CBL‐Y700 and CBL‐Y774. The findings demonstrated a significant reduction in phosphorylation at these sites following Tie2 suppression via shRNA, as illustrated in Figure 5I. Consequently, the phosphorylation of CBL was also reduced by kinase‐dead Tie2 (Myc‐Tie2‐L914F) (Figure S1V, Supporting Information). The endogenous interaction between CBL and PSAP was consistently diminished following Tie2 inhibition, as further corroborated by dual experimental analyses (refer to Figure S1N, Supporting Information). In reality, the phosphorylation of CBL itself enables its binding to Tie2 (Figure S1W, Supporting Information). Furthermore, the mutation of two phosphorylation sites led to a reduction in the binding affinity of CBL to PSAP (Figure S1S–U, Supporting Information). Therefore, the results suggest that the phosphorylation of CBL by Tie2 enhances the interaction between CBL and PSAP, leading to the stabilization of PSAP. Additionally, we discovered that the levels of Tie2 protein were substantially reduced in rat‐derived NP cells following the transfection of lentiviral shACE (Figure 5J). Moreover, the mutation of CBL phosphorylation sites resulted in a delayed Ub binding and a reduced rate of PSAP degradation, as illustrated in Figure 5K–M. It is noteworthy that the overexpression of Tie2 not only markedly decreased the expression of cellular senescence markers but also significantly mitigated degenerative characteristics in NP cells (Figure S1X, Supporting Information).

Moreover, our investigation unexpectedly revealed the critical function of the deubiquitinase Ub‐specific‐processing protease 20 (USP20) in the maintenance of PSAP protein homeostasis. The depletion of ACE markedly elevated the expression levels of USP20 (refer to Figure 5A). In contrast, the knockout of USP20 expedited the degradation of PSAP and enhanced its ubiquitination, as demonstrated in Figures 5N–P and Figure S1Y (Supporting Information). The discovery that USP20 competitively interacts with PSAP and CBL (Figure S1O, Supporting Information) is particularly intriguing, as it establishes a dynamic equilibrium that could provide an additional mechanism for the precise regulation of PSAP protein levels. These innovative discoveries significantly improve our comprehension of the regulatory network that orchestrates the stability of PSAP proteins.

### 2.6 RAW264.7 is Influenced by NP Cell‐Secreted PSAP Binding to GPR37

**Figure 6:**
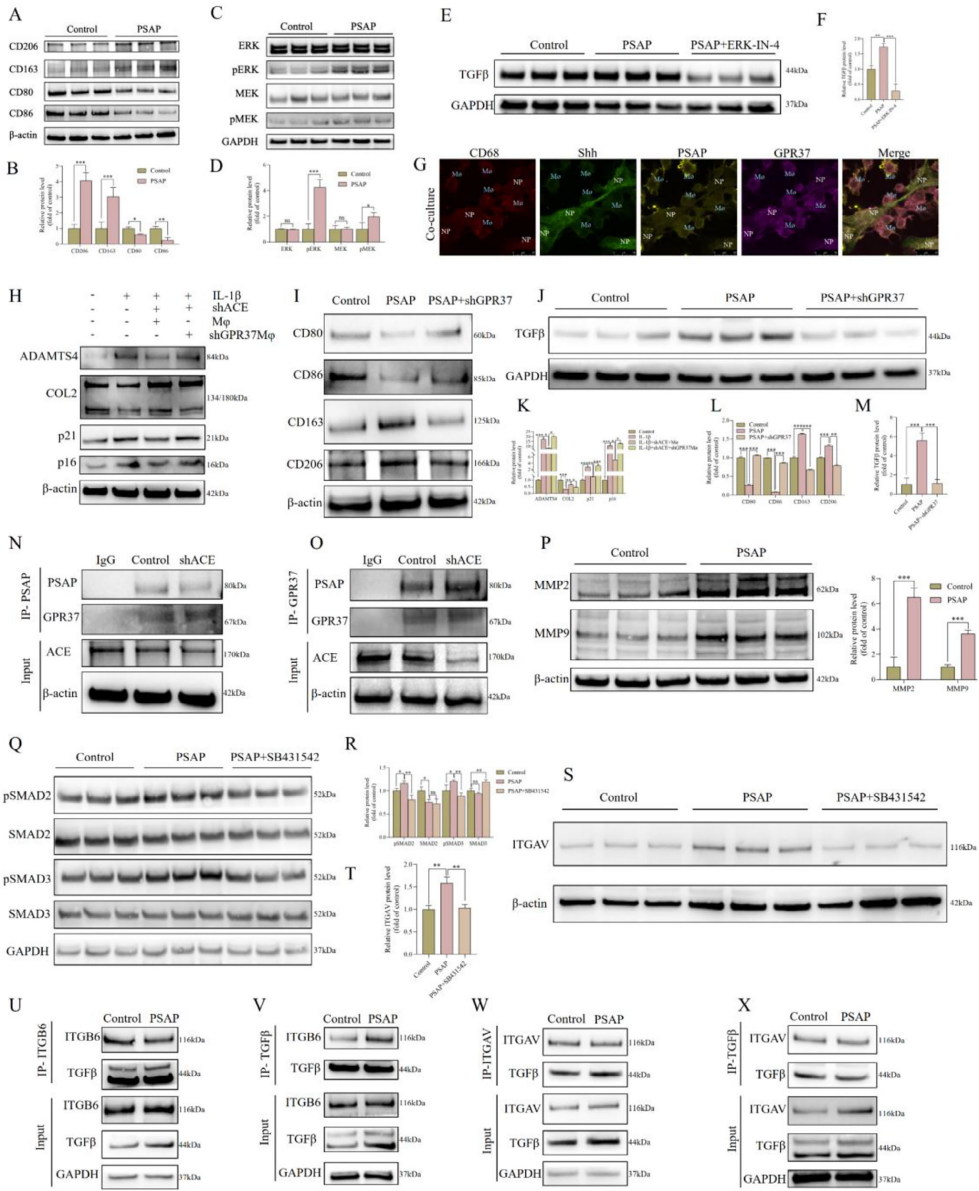
Mechanism study of PSAP secreted by SD rat‐derived NP cells regulating macrophage polarization. A,B) After PSAP recombinant protein (10 µm) treatment of RAW264.7 cells in vitro, M1 and M2 type macrophage marker protein levels were detected. Band gray values were semi‐quantitatively analyzed using ImageJ (n = 3). C,D) After PSAP recombinant protein (10 µm) treatment of RAW264.7 cells in vitro, total ERK/MEK protein and their phosphorylated protein levels were detected. Band gray values were semi‐quantitatively analyzed using ImageJ (n = 3). E,F) After treatment of RAW264.7 cells with PSAP recombinant protein (10 µm) or PSAP recombinant protein (10 µm) combined with ERK inhibitor ERK‐IN‐4 (50 µm) in vitro, TGFβ protein levels were detected. Band gray values were semi‐quantitatively analyzed using ImageJ (n = 3). G) For direct coculture, RAW264.7 and SD rat‐derived NP cells were co‐seeded at 1:1 ratio (1×10⁵ cells each) on collagen‐coated chamber slides, allowing direct cell‐cell interactions mimicking native tissue microenvironments before 48‐h confocal imaging with antibodies against CD68 (macrophages), PSAP, GPR37, and Shh (NP cells). This direct contact model enabled us to visualize cell morphology, cell fluorescence distribution, and cell interactions using confocal microscopy. H,K) Following IL‐1β (10 ng mL^−1^) stimulation of SD rat‐derived NP cells in vitro, we performed shACE (lentivirus carrying ACE shRNA) transfection and established a co‐culture system. The experimental setup consisted of either RAW264.7 cells or shGPR37 (lentivirus carrying GPR37 shRNA)‐transfected RAW264.7 cells seeded in the upper chamber of 0.4 µm pore Transwell inserts, while the transfected NP cells were maintained in the lower chamber. This configuration allowed for soluble factor exchange between cell populations without direct physical contact during the 48‐h incubation period. Subsequently, we assessed protein expression levels of degeneration markers (ADAMTS4 and COL2) and senescence markers (p16 and p21) in NP cells. Protein band intensities were quantified using ImageJ software for semi‐quantitative analysis (n = 3). I,L) After treatment of RAW264.7 cells with PSAP recombinant protein (10 µm) or PSAP recombinant protein (10 µm) combined with shGPR37 transfection in vitro, M1 and M2 type macrophage marker protein levels were detected. Band gray values were semi‐quantitatively analyzed using ImageJ (n = 3). J,M) After treatment of RAW264.7 cells with PSAP recombinant protein (10 µm) or PSAP recombinant protein (10 µm) combined with shGPR37 transfection in vitro, TGFβ protein levels were detected. Band gray values were semi‐quantitatively analyzed using ImageJ (n = 3). N,O) IB analysis of cell lysates and anti‐PSAP or anti‐GPR37 (as IP) derived from SD rat‐derived NP cells treated as indicated. All input lysates were subjected to IB for GPR37 and PSAP. P) After PSAP recombinant protein (10 µm) treatment of RAW264.7 cells in vitro, protein levels of TGFβ precursor cleavage enzymes MMP2 and MMP9 were detected. Band gray values were semi‐quantitatively analyzed using ImageJ (n = 3). Q,R) After treatment of RAW264.7 cells with PSAP recombinant protein (10 µm) or PSAP recombinant protein (10 µm) combined with TGFβ inhibitor SB431542 (10 µm) in vitro, total SMAD2/SMAD3 protein and their phosphorylated protein levels were detected. Band gray values were semi‐quantitatively analyzed using ImageJ (n = 3). S,T) After treatment of RAW264.7 cells with PSAP recombinant protein (10 µm) or PSAP recombinant protein (10 µm) combined with TGFβ inhibitor SB431542 (10 µm) in vitro, ITGAV protein levels were detected. Band gray values were semi‐quantitatively analyzed using ImageJ (n = 3). U,V) IB analysis of cell lysates and anti‐TGFβ or anti‐ITGB6 (as IP) derived from RAW264.7 cells treated as indicated. All input lysates were subjected to IB for ITGB6 and TGFβ. W,X) IB analysis of cell lysates and anti‐TGFβ or anti‐ITGAV (as IP) derived from RAW264.7 cells treated as indicated. All input lysates were subjected to IB for ITGAV and TGFβ. All data are expressed as the mean ± SD. Comparisons between two groups were made using the unpaired two‐tailed Student's t‐test, whereas comparisons among multiple groups were conducted using one‐way analysis of variance followed by Tukey's post hoc test. A *p*‐value of less than 0.05 was considered indicative of statistical significance. ^*^ *p* < 0.05, ^**^ *p* < 0.01, ^***^ *p* < 0.001, while “ns” indicates a lack of statistical significance. PSAP, Prosaposin; NP, Nucleus pulposus; ERK, Extracellular signal‐regulated kinase; MEK, Mitogen‐activated protein kinase; TGFβ, Transforming growth factor β; COL2, Collagen type II; CD68, Cluster of differentiation 68; GPR37, G protein‐coupled receptor 37; IL‐1β, Interleukin‐1β; ADAMTS4, A Disintegrin And Metalloproteinase with Thrombospondin Motifs 4; p16, Cyclin‐dependent kinase inhibitor 2A; p21, Cyclin‐dependent kinase inhibitor 1; IB, Immunoblotting; IP, Immunoprecipitation; MMP2, Matrix metalloproteinase‐2; MMP9, Matrix metalloproteinase‐9; SMAD2, Mothers against decapentaplegic homolog 2; SMAD3, Mothers against decapentaplegic homolog 3; ITGAV, Integrin alpha V; ITGB6, Integrin beta 6; Shh, Sonic hedgehog signaling molecule.

We conducted a systematic investigation into the regulatory effects of PSAP on macrophages and the associated molecular mechanisms in ACE knockout mice, with particular consideration given to the observed elevation in PSAP levels and the increased proportion of M2 macrophages. Initially, the treatment of RAW264.7 macrophages with recombinant PSAP protein significantly increased the expression of M2 markers (CD206 and CD163) and decreased the expression of M1 markers (**Figures** **6**A,B and S2A,C, Supporting Information). Additional transcriptome sequencing analysis between PSAP recombinant protein‐treated mouse‐derived RAW264.7 cells (Figure S3A–G, Supporting Information) and cell supernatant (Figure S3H–N, Supporting Information) demonstrated that the ERK signaling pathway is a critical regulator of this process, and there were substantial fluctuations in TGFβ levels, particularly in the supernatant (Figure S3A–N, Supporting Information). These results were corroborated by IF staining, quantitative analysis, and Wb (Figure 6C–F).

Confocal microscopy demonstrated that RAW264.7 and rat‐derived NP cells interacted through direct contact in coculture experiments, a process that was contingent upon the GPR37 receptor on the surfaces of RAW264.7 cells (Figure 6G). We must acknowledge that the current limitations of sonic hedgehog signaling molecule (Shh), CD68, PSAP, and GPR37 antibodies specificity currently on the market; nevertheless, we would like to emphasize that our cell identification was based on multiple robust criteria. Firstly, NP cells consistently exhibited a characteristic spindle‐shaped morphology with elongated cytoplasmic extensions, while macrophages displayed a distinct rounded/amoeboid shape – a well‐established morphological difference clearly visible in our high‐resolution confocal images. Importantly, we supplemented this morphological analysis with CD68 staining (a classic macrophage marker), which showed strong expression in the rounded cells membrane and weak positive on another cell membrane, providing additional confirmation of macrophage identity. However, in NP cells, the CD68 fluorescence signal was weak and uniformly distributed, lacking the characteristic membrane‐specific localization. The membrane‐localized GPR37 signal was consistently observed in these CD68‐positive, rounded macrophage membranes, while the positivity was weaker on the membranes of spindle‐shaped NP cells. Meanwhile, GPR37 is predominantly expressed in macrophages. This combined approach of morphological assessment (spindle vs rounded) and marker expression (CD68^+^ for macrophages) provides compelling evidence for accurate cell type discrimination. Simultaneously, we employed the Shh, a surface marker of NP cells, to label these cells.^[^ ^38^, ^39^ ^]^ Our observations indicate that, in contrast to the rounded morphology of macrophages, the irregular spindle‐shaped NP cells display pronounced positive staining for Shh. This distinct staining pattern effectively differentiates NP cells from macrophages. In order to further investigate the mechanistic function of GPR37 in macrophage regulation, we devised a series of rigorous experiments. Initially, we developed a model of rat‐derived NP cells degeneration that involved the use of IL‐1β treatment in conjunction with shACE intervention. Subsequently, we cocultured these treated NP cells with shGPR37‐transfected RAW264.7 cells. The findings demonstrated that GPR37 expression was suppressed in RAW264.7 cells, leading to a marked elevation in senescence markers and degeneration indicators in cocultured NP cells, as illustrated in Figures 6H,K and Figure S2B,D (Supporting Information). The critical function of GPR37 in the maintenance of NP cells homeostasis is clearly illustrated by this phenomenon. Further investigation demonstrated that the biological effects of PSAP were significantly reduced. Moreover, the function of RAW264.7 cells was affected by the knockout of GPR37, as illustrated in Figure 6I,L. In particular, the secreted TGFβ levels were significantly diminished, and PSAP‐induced M2 polarization was partially blocked in shGPR37‐transfected RAW264.7 cells (Figure 6J,M). These findings substantiate the role of GPR37 as the principal receptor for PSAP, which is crucial for modulating macrophage polarization and the secretion of TGFβ mediated by PSAP. PSAP binding to the GPR37 receptor was substantially enhanced by ACE knockdown in rat‐derived NP cells, as evidenced by subsequent experiments (Figure 6N,O). Furthermore, the interaction between PSAP and GPR37 was enhanced by elevated glycosylation levels (Figure S2U, Supporting Information). It is crucial to note that the expression of critical proteases matrix metalloproteinase‐2 (MMP2) and MMP9, which are involved in the cleavage of TGFβ precursors,^[^ ^40^, ^41^, ^42^, ^43^ ^]^ was substantially increased by PSAP treatment (Figure 6P). The findings suggest that PSAP potentially exerts its biological effects via two mechanisms: augmenting its binding affinity to the GPR37 receptor and facilitating the maturation and release of TGFβ.

PSAP modulates the phosphorylation of mothers against decapentaplegic homolog 2/3 (SMAD2/3) and enhances the expression of integrin subunit alpha V (ITGAV) in a manner dependent on TGFβ signaling, as evidenced by mechanistic studies (Figure 6Q–T). Considering the pivotal function of the ITGAV/integrin subunit beta 6 (ITGB6) complex in the transport and secretion of TGFβ, our study confirmed that PSAP treatment enhances the binding of TGFβ to ITGAV/ITGB6, consequently promoting the secretion of TGFβ (refer to Figure 6U–X). We identified three critical phosphorylation sites (Ser464, Ser465, and Ser467) that are essential for the PSAP‐SMAD interaction by constructing phosphorylation site mutants of SMAD2/3 (SMAD2‐3A and SMAD3‐3A) (Figure S2E–H, Supporting Information).

It is noteworthy that our findings indicate TGFβ can enhance the binding affinity of PSAP to SMAD2/3 in a concentration‐dependent manner, as depicted in Figure S2I–L (Supporting Information). Notably, the extent of PSAP glycosylation had a direct impact on the strength of its interactions with GPR37 and SMAD2/3. Elevated levels of glycosylation intensified these interactions, while mutations at glycosylation sites resulted in a reduction of interaction strength (Figure S2M–U, Supporting Information). Macrophages' secretion of TGFβ may function as a feedback mechanism to regulate macrophage polarization via the ERK pathway (Figure S3O, Supporting Information). Collectively, these findings reveal the complex molecular network by which PSAP precisely regulates macrophage polarization and TGFβ secretion.

### 2.7 NP Cells are Influenced by RAW264.7 via Secreted TGFβ

**Figure 7:**
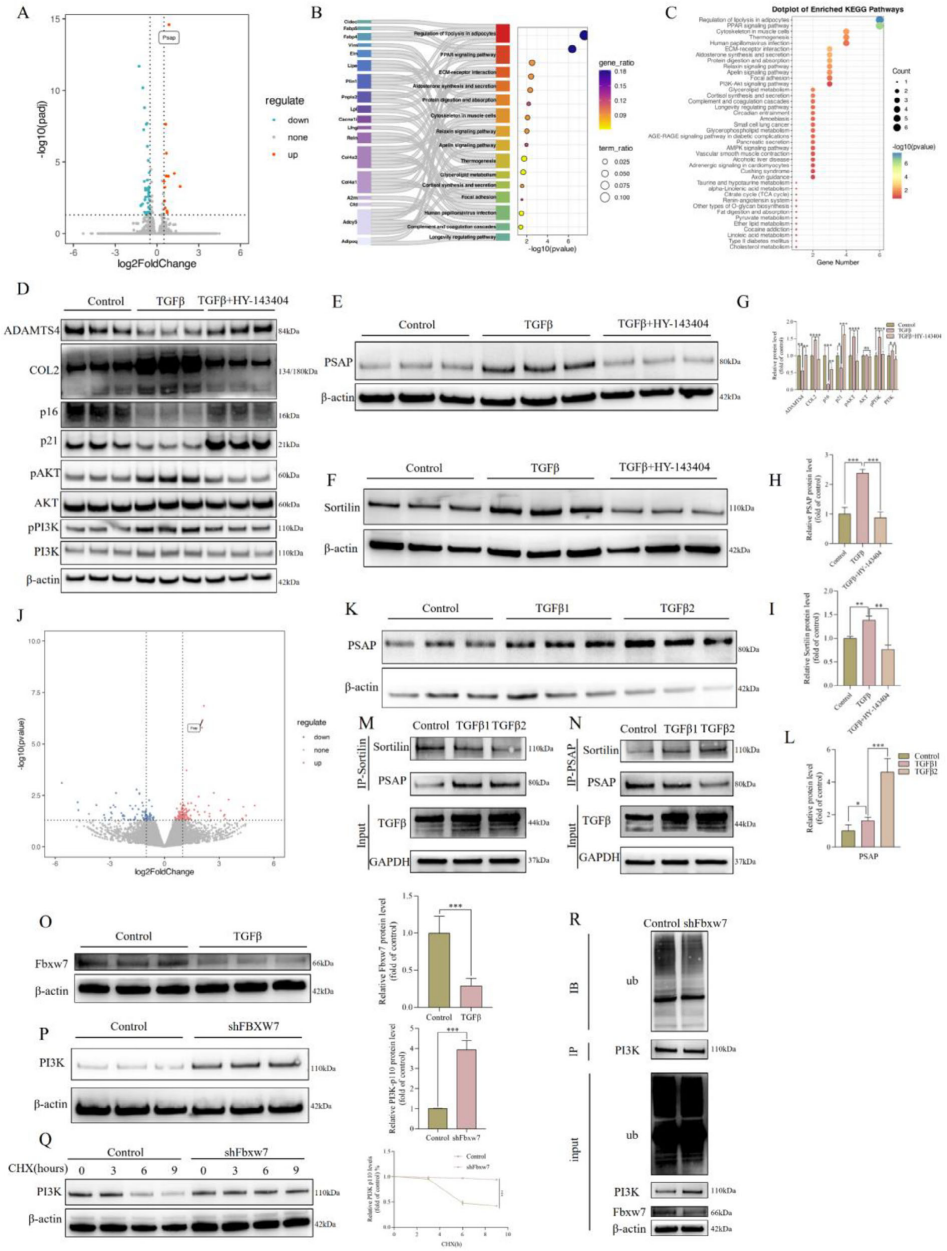
Mechanism study of TGFβ secreted by macrophages regulating NP cells degeneration. We performed transcriptome sequencing comparison between TGFβ recombinant protein (10 ng mL^−1^)‐treated rat‐derived NP cells and control groups (n = 3), followed by KEGG analyses of the differentially expressed genes. A) Volcano plots of differentially expressed gene between TGFβ recombinant protein (10 ng mL^−1^)‐treated rat‐derived NP cells and control groups. B,C) The Sankey and Bubble plot of metabolic pathway KEGG analysis. D,G) After treatment of SD rat‐derived NP cells with TGFβ recombinant protein (10 ng mL^−1^) or TGFβ recombinant protein (10 ng mL^−1^) combined with PI3K inhibitor HY‐143404 (10 µm) in vitro, degeneration (ADAMTS4 and COL2) and senescence (p16 and p21) related marker protein levels were detected. Band gray values were semi‐quantitatively analyzed using ImageJ (n = 3). E,H) After treatment of SD rat‐derived NP cells with TGFβ recombinant protein (10 ng mL^−1^) or TGFβ recombinant protein (10 ng mL^−1^) combined with PI3K inhibitor HY‐143404 (10 µm) in vitro, PSAP protein levels were detected. Band gray values were semi‐quantitatively analyzed using ImageJ (n = 3). F,I) After treatment of SD rat‐derived NP cells with TGFβ recombinant protein (10 ng mL^−1^) or TGFβ recombinant protein (10 ng mL^−1^) combined with PI3K inhibitor HY‐143404 (10 µm) in vitro, Sortilin protein levels were detected. Band gray values were semi‐quantitatively analyzed using ImageJ (n = 3). J) Differential gene analysis of supernatant after TGFβ recombinant protein (10 ng mL^−1^) treatment of SD rat‐derived NP cells in vitro. K,L) After treatment of SD rat‐derived NP cells with different concentrations of TGFβ recombinant protein (concentration #1‐5 ng mL^−1^ or concentration #2‐10 ng mL^−1^) in vitro, PSAP protein levels were detected (n = 3). M,N) IB analysis of cell lysates and anti‐Sortilin or anti‐PSAP (IP) derived from SD rat‐derived NP cells treated as indicated. The TGFβ protein concentration used was as follows (concentration #1: 5 ng mL^−1^ or concentration #2: 10 ng mL^−1^). All input lysates were subjected to IB for Sortilin and PSAP. O) After TGFβ recombinant protein (10 ng mL^−1^) treatment of SD rat‐derived NP cells in vitro, Fbxw7 protein levels were detected. Band gray values were semi‐quantitatively analyzed using ImageJ (n = 3). P) After shFbxw7 (lentivirus carrying Fbxw7 shRNA) transfection of SD rat‐derived NP cells in vitro, PI3K protein levels were detected. Band gray values were semi‐quantitatively analyzed using ImageJ (n = 3). Q) SD rat‐derived NP cells transfected with shFbxw7 were treated with CHX (50 µg mL^−1^) for specified durations, followed by IB of cell lysates to determine the relative levels of PI3K compared to β‐actin (n = 3). In the quantitative line graph, the x‐axis represents the CHX (50 µg mL^−1^) treatment time, while the y‐axis indicates the relative expression level of the target protein normalized to β‐actin (with the 0‐h value set as 100%). R) SD rat‐derived NP cells underwent transfection with shFbxw7 for a duration of 72 h. The cell lysate underwent IP using PI3K antibody, then followed by IB using Ub antibody. All data are expressed as the mean ± SD. Comparisons between two groups were made using the unpaired two‐tailed Student's *t*‐test, whereas comparisons among multiple groups were conducted using one‐way analysis of variance followed by Tukey's post hoc test. A *p*‐value of less than 0.05 was considered indicative of statistical significance. ^*^ *p* < 0.05, ^**^ *p* < 0.01, ^***^ *p* < 0.001, while “ns” indicates a lack of statistical significance. TGFβ, Transforming growth factor β; NP, Nucleus pulposus; GO, Gene Ontology; KEGG, Kyoto Encyclopedia of Genes and Genomes; PI3K, Phosphoinositide 3‐kinase; ADAMTS4, A Disintegrin And Metalloproteinase with Thrombospondin Motifs 4; COL2, Collagen type II; p16, Cyclin‐dependent kinase inhibitor 2A; p21, Cyclin‐dependent kinase inhibitor 1; PSAP, Prosaposin; IB, Immunoblotting; IP, Immunoprecipitation; Fbxw7, F‐box/WD repeat‐containing protein 7; CHX, Cycloheximide; Ub, Ubiquitin.

We conducted a more thorough investigation of the regulatory effects of TGFβ on rat‐derived NP cells, building on our previous discoveries that PSAP promotes TGFβ secretion from RAW264.7 macrophages. The transcriptionome sequencing comparison analysis between TGFβ recombinant protein‐treated rat‐derived NP cells and normal control groups (Figure S4A–D, Supporting Information) revealed a substantial increase in PSAP expression, with pathway enrichment analysis emphasizing the critical role of the PI3K/AKT signaling pathway (**Figure** **7**A–C). This discovery was corroborated by subsequent Wb and IF experiments, which demonstrated that TGFβ treatment did, in fact, alleviate NP cells degeneration and senescence phenotypes in a concentration‐dependent manner while promoting PSAP expression. The PI3K inhibitor HY‐143404 partially reversed these protective effects (Figure 7D–E,G,H, Figure S4E–H, Supporting Information). We observed that the expression pattern of the PSAP transporter Sortilin mirrored that of PSAP in the TGFβ regulatory network. This is particularly noteworthy. Specifically, the expression of Sortilin was significantly upregulated following TGFβ stimulation; however, this effect was reversed upon administration of HY‐143404, as illustrated in Figure 7F,I. Transcriptome sequencing data from the supernatants of NP cells further corroborated this regulatory relationship, as the increased levels of PSAP were entirely consistent with the previously mentioned findings (refer to Figure 7J–L; additional information is available in Figure S4I–M, Supporting Information). More importantly, the TGFβ‐PSAP‐Sortilin regulatory axis was directly demonstrated by the dose‐dependent enhancement of PSAP binding to Sortilin as TGFβ stimulation concentrations increased (Figure 7M,N).

For the purpose of elucidating the molecular mechanism by which TGFβ regulates the PI3K/AKT pathway, we compared transcriptome data from rat‐derived NP cells before and after TGFβ treatment and identified a substantial decrease in the activity of the E3 Ub ligase F‐box and WD repeat domain containing 7 (Fbxw7). The protein level of this result was verified by Wb (Figure 7O). Functional analyses revealed that the knockout of Fbxw7 led to an elevation in PI3K protein levels, a marked reduction in PI3K ubiquitination, and a deceleration in its degradation rate (Figure 7P–R). Collectively, these findings elucidate the specific regulatory mechanism through which TGFβ stabilizes PI3K protein by downregulating Fbxw7, consequently activating the PI3K/AKT signaling pathway.

### 2.8 Animal Studies

**Figure 8:**
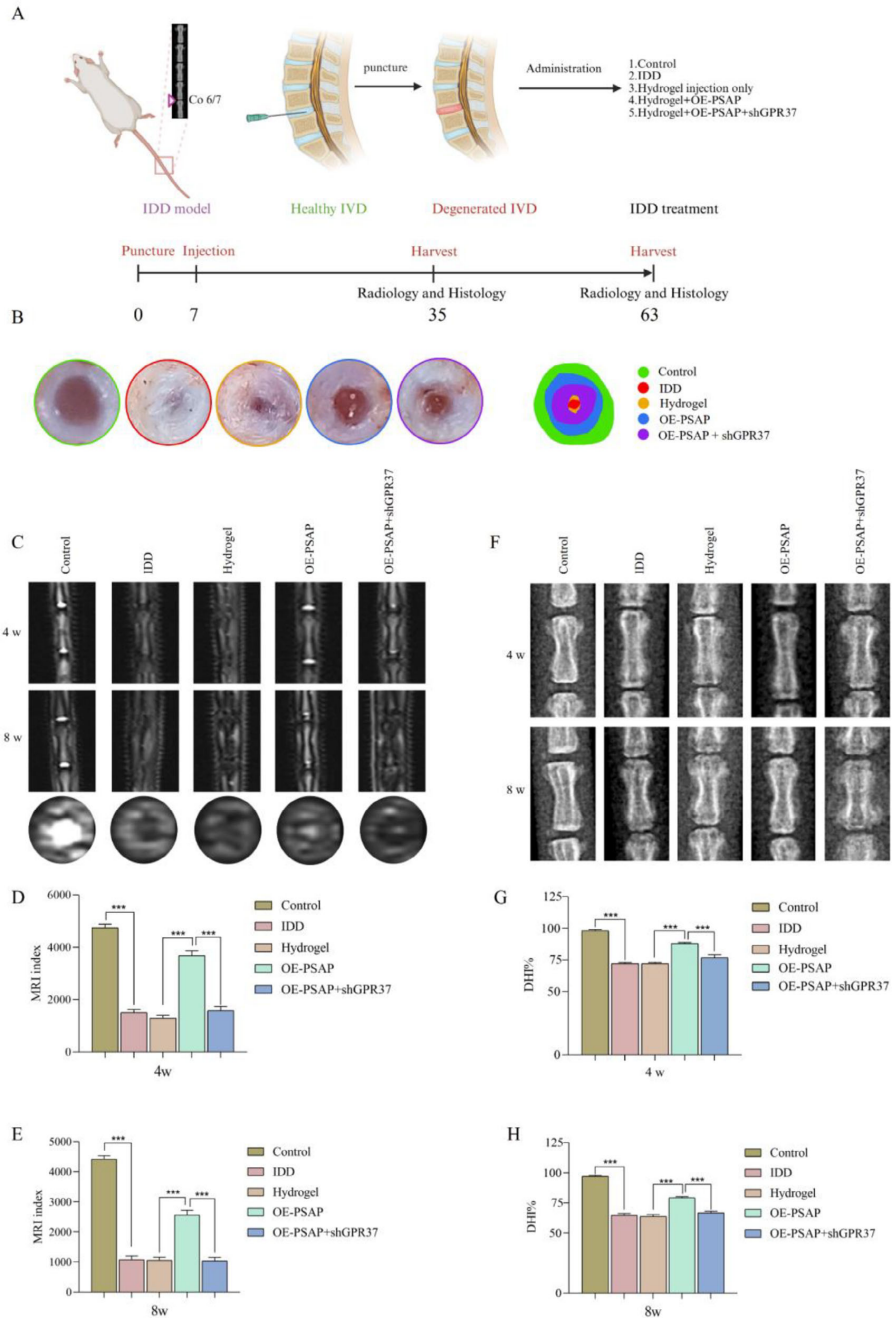
Macroscopic observation of IVD and evaluation of rat tail X‐ray and MRI. A) Schematic diagram of animal experiment. B) Macroscopic images of IVD, fitting diagram and relative NP area at 8 weeks after intervention. C–E) Representative MRI scans and their MRI index calculation obtained at 4 and 8 weeks after intervention (n = 6). Disc rehydration was quantified using the MRI index, calculated as the product of NP area and signal intensity derived from midsagittal T2‐weighted images. Higher index values indicate better tissue hydration and proteoglycan content, consistent with quantitative MRI assessments of disc degeneration. F–H) Representative X‐ray images of SD rat tails and their DHI quantitative calculation at 4 and 8 weeks after intervention (n = 6). DHI% was calculated as: (D+E+F) × 100%/(A+B+C+D+E+F). A, B, and C are the heights of the anterior, midpoint, and posterior edge of the upper vertebral body, respectively. D, E, and F are the heights of the anterior, midpoint, and posterior edge of the intervertebral space, respectively. All data are expressed as mean ± SD. Comparisons between two groups were made using the unpaired two‐tailed Student's *t*‐test, whereas comparisons among multiple groups were conducted using one‐way analysis of variance followed by Tukey's post hoc test. A *p*‐value of less than 0.05 was considered indicative of statistical significance. ^*^ *p* < 0.05, ^**^ *p* < 0.01, ^***^ *p* < 0.001, while “ns” indicates a lack of statistical significance. IVD, Intervertebral disc; NP, Nucleus pulposus; DHI, Disc height index; MRI, Magnetic resonance imaging.

The experimental flowchart is depicted in **Figure** **8**A. Initially, we conducted morphological evaluations of IVDs (Figure 8B). Macroscopic observations revealed that both the control and hydrogel+Lv‐OE‐PSAP groups preserved a consistent IVD architecture, characterized by well‐defined boundaries between the NP and annulus fibrosus (AF), as well as substantial hydration. Conversely, the IDD, hydrogel, and hydrogel+Lv‐OE‐PSAP+AAV‐shGPR37 groups demonstrated characteristic degenerative features, including a disorganized IVD structure, a diminished NP volume, and a thickened AF. Quantitative analysis indicated that the hydrogel+Lv‐OE‐PSAP group demonstrated a significantly larger relative NP area compared to the other treatment groups.

The imaging evaluation results indicated that the MRI index analysis further confirmed that the hydrogel+Lv‐OE‐PSAP group exhibited substantially less hydration loss at 4 and 8 weeks than other treatment groups (except the control group) (Figure 8C–E). The hydrogel+Lv‐OE‐PSAP group exhibited a significantly higher disc height index (DHI) at 4 and 8 weeks compared to the IDD, hydrogel, and hydrogel+Lv‐OE‐PSAP+AAV‐shGPR37 groups in X‐ray examinations (Figure 8F–H).

**Figure 9:**
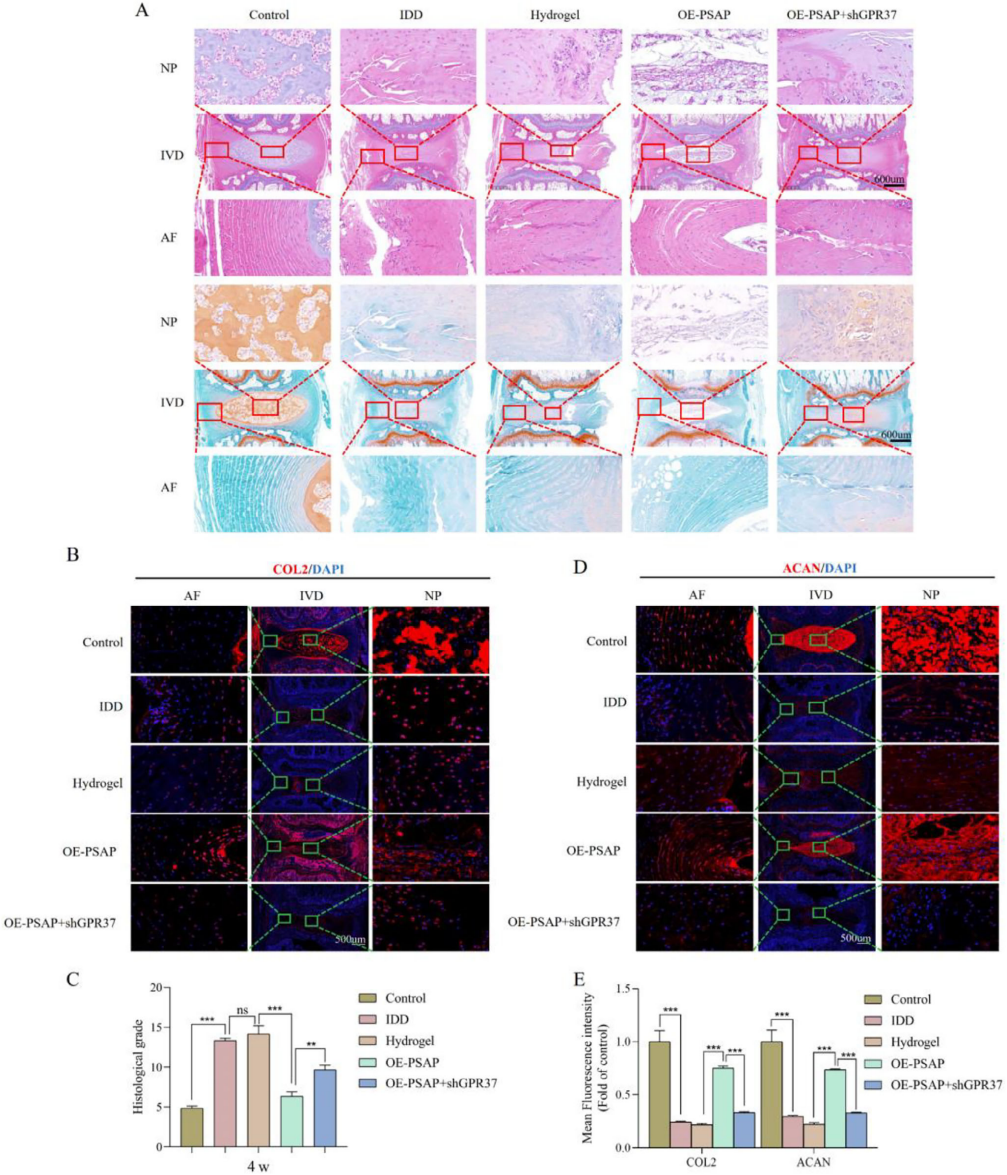
Effects of virus‐loaded composite hydrogel on histological scores and ECM of rat tailbone IVD corresponding to 4 weeks after intervention. A) Representative HE staining and SO staining images (coronal position) of SD rat tailbone IVD corresponding to different treatment groups (n = 6). B–E) COL2 and ACAN IF staining detection and quantitative analysis of IF staining at 4 weeks after intervention (n = 6). All data are expressed as mean ± SD. Comparisons between two groups were made using the unpaired two‐tailed Student's *t*‐test, whereas comparisons among multiple groups were conducted using one‐way analysis of variance followed by Tukey's post hoc test. A *p*‐value of less than 0.05 was considered indicative of statistical significance. ^*^ *p* < 0.05, ^**^ *p* < 0.01, ^***^ *p* < 0.001, while “ns” indicates a lack of statistical significance. ECM, Extracellular matrix; IVD, Intervertebral disc; HE, Hematoxylin and eosin; SO, Safranine O‐Fast Green; COL2, Collagen type II; ACAN, Aggrecan; IF, Immunofluorescence.

**Figure 10:**
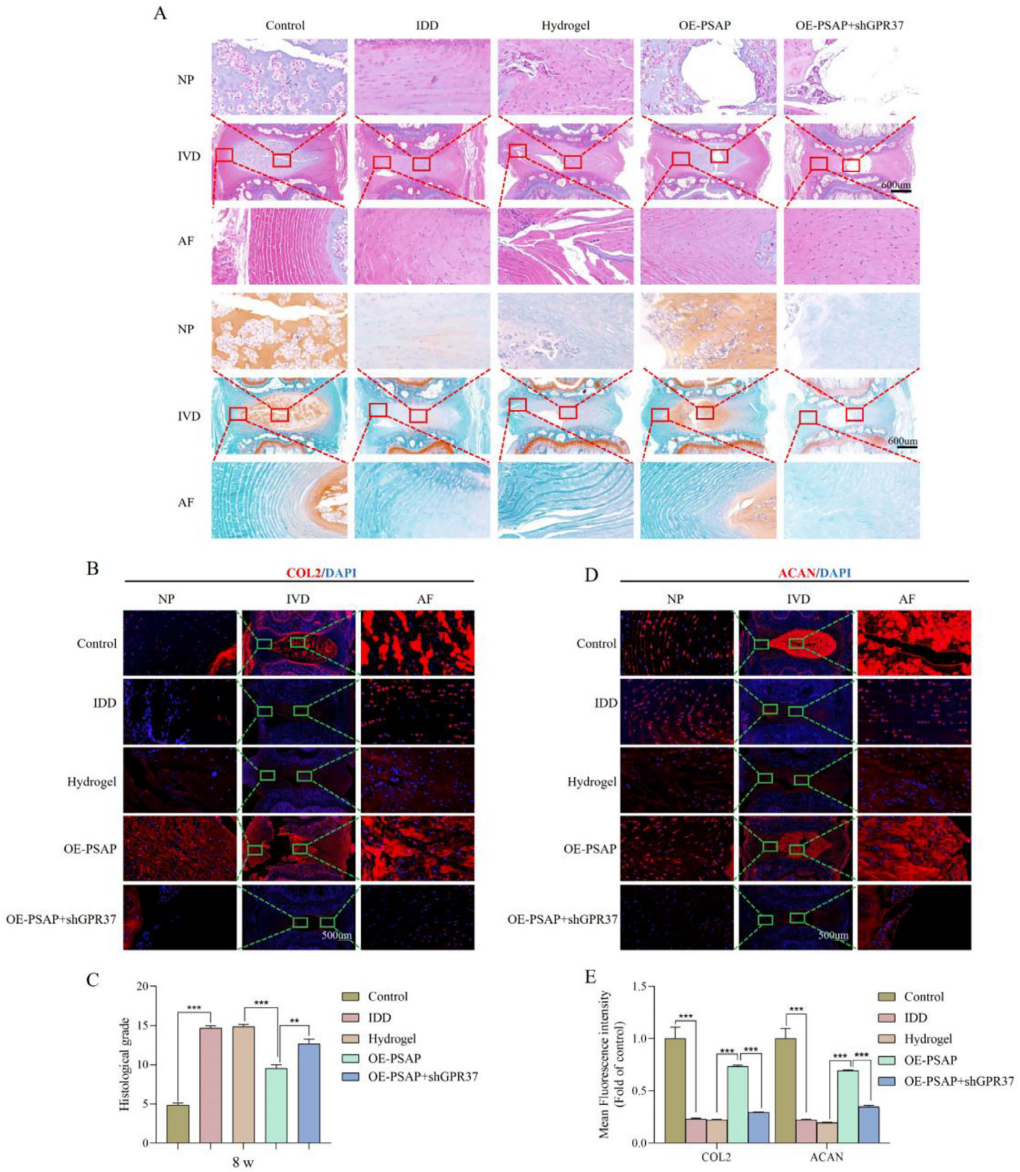
Effects of virus‐loaded composite hydrogel on histological scores and ECM of rat tailbone IVD corresponding to 8 weeks after intervention. A) Representative HE staining and SO staining images (coronal position) of SD rat tailbone IVD corresponding to different treatment groups (n = 6). B–E) COL2 and ACAN IF staining detection and quantitative analysis of IF staining at 8 weeks after intervention (n = 6). All data are expressed as mean ± SD. Comparisons between two groups were made using the unpaired two‐tailed Student's *t*‐test, whereas comparisons among multiple groups were conducted using one‐way analysis of variance followed by Tukey's post hoc test. A *p*‐value of less than 0.05 was considered indicative of statistical significance. ^*^ *p* < 0.05, ^**^ *p* < 0.01, ^***^ *p* < 0.001, while “ns” indicates a lack of statistical significance. ECM, Extracellular matrix; IVD, Intervertebral disc; HE, Hematoxylin and eosin; SO, Safranine O‐Fast Green; COL2, Collagen type II; ACAN, Aggrecan; IF, Immunofluorescence.

Histological analysis revealed that the hydrogel combined with Lv‐OE‐PSAP group preserved the partial NP tissues structure, as evidenced by HE staining at both 4 and 8 weeks. This group demonstrated significantly superior tissue preservation compared to the other treatment groups, as illustrated in **Figures** **9**A and **10**A. The proteoglycan loss in this cohort was significantly lower than that of other treatments, as evidenced by SO staining. The hydrogel+Lv‐OE‐PSAP group maintained higher expression levels of collagen type II (COL2) and aggrecan (ACAN) at both 4 and 8 weeks in comparison to other treatment groups, as confirmed by IF detection (Figure 9B–E and Figure 10B–E). In addition, regarding macrophage infiltration, we found that compared with the IDD group, the expression of the M2 macrophage marker (CD163) was significantly increased, while the expression of the M1 macrophage marker (CD86) was significantly decreased after overexpression of PSAP (Figure S5, Supporting Information). However, this phenomenon was reversed after subsequent specific knockout of GPR37 in macrophages.

## 3 Discussion

In this study, we revealed the crucial role of ACE knockdown in regulating PSAP metabolism in NP cells. Our findings demonstrate that ACE knockdown significantly enhances PSAP glycosylation modification. This increased glycosylation promotes PSAP secretion to the cell membrane and ECM by reducing CBL‐mediated ubiquitination and subsequent degradation. The elevated PSAP levels effectively alleviate IL‐1β‐induced NP cells senescence and degeneration phenotypes through activation of the PI3K/AKT signaling pathway. Importantly, we discovered that PSAP secreted by NP cells specifically binds to GPR37 on macrophage surfaces. By activating ERK‐related signaling pathways, PSAP promotes macrophage polarization toward the anti‐inflammatory M2 phenotype. The TGFβ secreted by M2 macrophages further feeds back to NP cells, forming a positive regulatory loop of increased PSAP secretion‐enhanced glycosylation‐augmented secretion. This discovery not only elucidates the critical regulatory role of the ACE‐PSAP‐GPR37 axis in IDD but also provides an important theoretical foundation for developing therapeutic strategies targeting PSAP metabolic pathways.

Our results demonstrate that ACE deficiency enhances the glycosylation of PSAP, thereby stabilizing it against proteasomal degradation. This finding is significant because it highlights the role of ACE in regulating PSAP metabolism. ACE is a well‐known enzyme involved in the renin‐angiotensin system, and its impact on glycosylation has not been extensively explored in the context of IDD. Our study shows that ACE knockdown leads to increased O‐GlcNAc modification of PSAP, which in turn prevents its ubiquitination by the E3 Ub ligase CBL. This mechanism is consistent with previous studies demonstrating that glycosylation can protect proteins from degradation by altering their conformation or reducing their affinity for E3 ligases.^[^ ^44^, ^45^, ^46^, ^47^ ^]^ The stabilization of PSAP is crucial for its biological functions, including its role in promoting anti‐inflammatory M2 macrophage polarization and TGFβ secretion. Our study reveals that PSAP glycosylation is not only important for its stability but also for its ability to interact with GPR37 on macrophages. This interaction is essential for the downstream signaling events that promote disc health. Previous studies have shown that PSAP can activate ERK and PI3K‐AKT signaling pathways, which are involved in cell survival and anti‐inflammatory responses.^[^ ^11^, ^14^, ^15^, ^48^, ^49^ ^]^ Our findings extend this knowledge by demonstrating that ACE‐mediated glycosylation is a key regulatory step in PSAP function.

As a secretory protein, PSAP can be secreted into the extracellular space, where it exerts diverse biological functions. Its secretion process is strictly regulated by multiple factors to ensure its proper physiological function.^[^ ^50^ ^]^ In other words, PSAP secretion depends not only on its synthesis and processing but also on intracellular signaling pathways and extracellular environmental cues.^[^ ^50^ ^]^ For example, under cellular stress conditions such as oxidative stress or nerve injury, PSAP secretion is significantly increased.^[^ ^51^ ^]^ This enhanced secretion may be related to PSAP's role in cytoprotecting and tissue repair.^[^ ^48^ ^]^ Sortilin is a key sorting receptor for PSAP, regulating its trafficking and secretion.^[^ ^33^ ^]^ Sortilin has been identified as a critical sorting receptor for PSAP, as cells lacking sortilin exhibit impaired PSAP precursor transport to lysosomal compartments.^[^ ^34^ ^]^ Furthermore, PSAP secretion may be regulated by intracellular signaling pathways, such as ERK and PI3K‐AKT pathway activation, which can influence PSAP secretion levels.^[^ ^52^ ^]^ For instance, in various cell types, PSAP secretion can be modulated through ERK signaling, thereby affecting cellular physiological functions. In this study, we found that after ACE knockdown, the binding capacity of Sortilin to PSAP was enhanced, which is conducive to the transport of PSAP to the cell membrane and outside the cell for corresponding physiological activities. Not only that, but also the increased glycosylation degree of PSAP caused by ACE knockdown is not only beneficial for the protein stability of the latter, but also more conducive to the further interaction between the latter and Sortilin. This point has been verified not only at the protein level, but also through dynamic modification proteomics and molecular docking simulations. In addition, PSAP secreted by NP cells subsequently acts on macrophages to promote the secretion of TGFβ by the latter, and TGFβ in turn promotes the increase of Sortilin protein levels in NP cells and the interaction strength with PSAP protein, thereby enhancing the functional intensity of PSAP and forming a positive feedback loop. Moreover, our study highlights the importance of glycosylation in modulating protein–protein interactions. The enhanced glycosylation of PSAP not only prevents its degradation but also facilitates its binding to Sortilin, thereby enhancing its signaling capacity. This finding is supported by previous research showing that glycosylation can modulate receptor‐ligand interactions and downstream signaling events.^[^ ^53^, ^54^ ^]^ Our results suggest that the glycosylation status of PSAP is a critical determinant of its biological activity, and manipulating this post‐translational modification could have therapeutic potential in IDD. The interaction between PSAP and GPR37 is a central mechanism in our study. We found that PSAP specifically binds to GPR37 on macrophages, promoting their polarization toward the anti‐inflammatory M2 phenotype. This is supported by previous studies demonstrating that PSAP‐GPR37 signaling enhances macrophage phagocytic function and reduces the secretion of pro‐inflammatory cytokines.^[^ ^22^, ^55^ ^]^ Our results further show that TGFβ secreted by M2 macrophages enhances PSAP secretion and glycosylation in NP cells, forming a positive feedback loop. This loop is critical for maintaining disc homeostasis and reducing inflammation.

The role of GPR37 in immune regulation has been previously highlighted in various contexts, including neurodegenerative diseases and infection models.^[^ ^56^ ^]^ GPR37 is involved in multiple physiological pathways, such as the resolution of inflammatory pain and oligodendrocyte differentiation. Acts as a receptor for diverse ligands, including osteocalcin (OCN) or neuroprotectin D1. Ligand binding induces endocytosis followed by an ERK phosphorylation cascade.^[^ ^57^, ^58^ ^]^ As a receptor for OCN, it regulates oligodendrocyte differentiation and myelination in the central nervous system.^[^ ^59^ ^]^ Our study adds to this body of literature by demonstrating that GPR37 is essential for PSAP‐mediated macrophage polarization and TGFβ secretion in IDD. In our study, we established a model of NP cell degeneration derived from rats, utilizing IL‐1β treatment in combination with shACE intervention. Subsequently, these treated NP cells were cocultured with RAW264.7 cells transfected with shGPR37. The findings demonstrated that GPR37 expression was suppressed in RAW264.7 cells, leading to a marked increase in senescence markers and degeneration indicators in the cocultured NP cells. This phenomenon underscores the essential role of GPR37 in maintaining NP cells homeostasis. Furthermore, in animal experiments concerning macrophage infiltration, our findings indicated a significant upregulation in the expression of the M2 macrophage marker (CD163) and a notable downregulation of the M1 marker (CD86) following the overexpression of PSAP. This effect, however, was reversed upon the targeted knockout of GPR37 in macrophages. This finding underscores the importance of targeting the PSAP‐GPR37 axis for therapeutic interventions. However, it is important to note that GPR37 is an orphan receptor with multiple potential ligands, and its role in different tissues and diseases may vary. Future studies should explore the specificity and selectivity of PSAP‐GPR37 interactions in the context of IDD. Furthermore, our study provides insights into the molecular mechanisms underlying PSAP‐GPR37‐mediated macrophage polarization. We found that the binding of PSAP to GPR37 activates ERK signaling pathways, which are crucial for promoting the M2 phenotype. This is consistent with previous studies showing that ERK signaling can modulate macrophage polarization and inflammatory responses.^[^ ^60^, ^61^ ^]^ Additionally, our results highlight the importance of TGFβ in maintaining the positive feedback loop between PSAP and macrophages. This loop not only enhances disc health but also reinforces the anti‐inflammatory environment within the IVD.

TGFβ is a pleiotropic cytokine with critical roles in tissue repair and inflammation modulation.^[^ ^62^ ^]^ Similarly, this study also found that TGFβ can regulate the degeneration and aging of NP cells by affecting the PI3K/AKT pathway. Furthermore, we conducted an in‐depth exploration of the potential mechanisms involved. The findings indicated that treatment of NP cells with TGFβ resulted in a reduction of Fbxw7 levels, which is implicated in the ubiquitination and degradation of PI3K. This observation partially elucidates the regulatory mechanism by which TGFβ influences PI3K. In addition, our study shows that TGFβ secreted by M2 macrophages enhances PSAP expression and glycosylation in NP cells, forming a positive feedback loop that promotes disc health. This finding is consistent with previous studies^[^ ^63^, ^64^ ^]^ demonstrating that TGFβ can upregulate PSAP expression and glycosylation, highlighting the importance of this axis in tissue repair and inflammation modulation. The role of TGFβ in IDD has been previously explored, with studies showing that TGFβ signaling can promote ECM synthesis and inhibit NP cells apoptosis.^[^ ^65^, ^66^ ^]^ Our results extend this knowledge by demonstrating that TGFβ signaling is enhanced through the PSAP‐GPR37 axis. This finding suggests that targeting this axis could have therapeutic benefits for IDD by promoting disc repair and reducing inflammation. However, it is important to note that TGFβ signaling is complex and context‐dependent, and its effects may vary depending on the stage of IDD and the specific cellular environment. Future studies should explore the temporal and spatial dynamics of TGFβ signaling in IDD to better understand its therapeutic potential. Moreover, our study highlights the importance of TGFβ in maintaining the positive feedback loop between PSAP and macrophages. TGFβ enhances PSAP secretion, and the latter promotes the expression of integrin subunits (ITGAV and ITGB6), which are crucial for TGFβ binding and secretion. This finding is supported by previous studies showing that TGFβ can modulate its own secretion through interactions with integrin subunits.^[^ ^66^, ^67^ ^]^ In addition, MMPs such as MMP2/9 are well‐documented activators of latent TGFβ through proteolytic cleavage of its binding proteins.^[^ ^42^, ^43^ ^]^ Our results (Figures 6P–X) demonstrate that PSAP treatment significantly upregulates MMP2/9 expression, thereby promoting TGFβ activation. Importantly, we identified that PSAP exerts its effects on TGFβ activation through two interconnected mechanisms: First, PSAP directly enhances MMP2/9 expression via the ERK/SMAD2/3 pathway, as evidenced by our phosphorylation assays (Figures 6Q–T). Second, PSAP modulates the TGFβ‐SMAD2/3 signaling axis by altering the phosphorylation status of SMAD2/3. Specifically, our data show that PSAP treatment increases phosphorylation of SMAD2/3 at critical serine residues (Ser464/465/467), which enhances their nuclear translocation and transcriptional activity.^[^ ^68^ ^]^ This dual regulation creates a robust positive feedback loop: activated TGFβ further reinforces PSAP secretion through PI3K/AKT pathway activation (Figures 7A–N), while simultaneously maintaining elevated MMPs activity to sustain TGFβ activation. As recently discussed in a comprehensive review on ECM remodeling,^[^ ^69^ ^]^ such intricate crosstalk between growth factors and MMPs represents a fundamental mechanism in tissue homeostasis.

While our study provides significant insights into the ACE‐PSAP‐GPR37 axis in IDD, there are limitations that need to be addressed. First, while our rodent models provide valuable mechanistic insights, we acknowledge that differences in disc anatomy and aging processes between rodents and humans may affect the translational relevance of our findings. Future studies should consider using more clinically relevant models, such as those involving genetic mutations or aging‐related IDD, to validate our findings. Second, our study focused on the role of PSAP glycosylation in macrophage polarization and TGFβ signaling, but other potential downstream pathways and interactions may also be involved in IDD pathogenesis. For example, PSAP can interact with other receptors, such as the low‐density lipoprotein receptor‐related protein (LRP), which may also play roles in IDD.^[^ ^70^, ^71^, ^72^, ^73^ ^]^ Future work should aim to comprehensively map the signaling networks regulated by PSAP in IDD to identify additional therapeutic targets. Thirdly, as mentioned earlier, the current limitations of non‐specific staining in existing antibodies hinder the demonstration of direct interactions between NP cells and macrophages. In the future, we will employ more advanced antibodies and techniques, potentially utilizing single‐cell sequencing to further explore the interaction mechanisms between these two cell types. Finally, although we demonstrate that ACE deficiency enhances OGT‐mediated PSAP glycosylation, the precise molecular mechanism linking ACE to OGT regulation remains incompletely characterized and warrants further investigation. Based on existing literature,^[^ ^10^, ^74^ ^]^ we hypothesize that ACE primarily regulates OGT at the protein level, not through transcription. OGT protein stability is controlled by the Ub‐proteasome system, with E3 ligases like β‐transducin repeat containing E3 Ubiquitin Protein Ligase and X‐linked inhibitor of apoptosis facilitating its degradation.^[^ ^75^, ^76^ ^]^ Additionally, OGT's stability is affected by its interaction with molecular chaperones like heat shock protein 90 (Hsp90), as inhibiting Hsp90 leads to OGT degradation.^[^ ^77^ ^]^ These mechanisms influence OGT protein levels in an ACE‐deficient environment. While evidence strongly supports ACE's main role in regulating OGT protein stability, we cannot completely rule out its indirect influence on OGT transcription through other pathways. This is because OGT transcription can be affected by factors like NFE2, like BZIP transcription factor 2,^[^ ^78^ ^]^ and ACE might impact these factors via PI3K/AKT and ERK pathways based on our findings and previous literature.^[^ ^10^, ^79^, ^80^, ^81^ ^]^ This complex regulation suggests that future research should use integrated multi‐omics analyses, precise OGT activity measurements, and structure‐function studies to fully understand the ACE‐OGT regulatory network.

## 4 Conclusion

In conclusion, our study provides novel insights into the regulatory mechanisms of PSAP stability and its role in IDD. We have demonstrated that ACE knockdown‐mediated glycosylation is critical for PSAP function and identified the PSAP‐GPR37 axis as a key regulator of macrophage polarization and TGFβ signaling to regulate NP cell degeneration. These findings not only advance our understanding of IDD pathogenesis but also offer a potential therapeutic strategy for its treatment.

## 5 Experimental Section

### Clinical Sample Collection

All NP tissues were obtained from patients undergoing necessary surgical procedures at the hospital. Degenerated NP samples were collected from 6 IDD patients, while normal NP tissues were acquired from 3 non‐IDD patients with fracture‐related neurological deficits. Three spinal surgeons independently evaluated IDD severity using MRI according to the Pfirrmann grading system (see Table S1, Supporting Information for detailed sample information). Pfirrmann grade II and V samples were selected for pathological staining, while portions of grades II, III, and V NP tissues were reserved for Wb analysis. This study received approval from the Tongji University Ethics Committee (Approval No. TJBB05022101). Informed consent is taken from all the participants present in the study. All procedures were strictly followed by the Declaration of Helsinki guidelines.

### Cell Isolation and Culture

All cellular experiments were performed using NP cells isolated from 1‐month‐old Sprague‐Dawley (SD) rats and the mouse‐derived macrophage cell line RAW264.7. NP cells were isolated from 1‐month‐old SD rats euthanized with sodium pentobarbital (50 mg kg^−1^). After dissecting the lumbar IVDs, NP tissues were digested with 0.1% collagenase II for 4 h at 37 °C. The cell suspension was filtered through 200‐µm mesh and cultured in complete DMEM/F12 medium supplemented with 10% fetal bovine serum (FBS) (A5256701, Gibco) and 1% penicillin/streptomycin (15 070 063, Gibco). Cells were maintained at 37 °C with 5% CO_2_, with medium changes every 3 days. RAW264.7 cells (CBP60533, Cell Bank of Shanghai Academy of Chinese Sciences) were cultured under identical conditions to NP cells. Second‐passage cells at 80–90% confluency were used for experiments.

### Cell Treatments and Transfection

For in vitro IDD modeling, NP cells were treated with 10 ng mL^−1^ IL‐1β (RIL1BI, Thermo Fisher) or 10 ng mL^−1^ TNF‐α (HY‐P70800, MedChemExpress) for 72 h. Various treatments were applied including: shRNA transfection (targeting CBL, USP20, or ACE), recombinant proteins (PSAP (10 µm, HY‐P77159, MedChemExpress, duration: 48 h) or TGFβ (concentration #1‐5 ng mL^−1^ or concentration #2–10 ng mL^−1^, HY‐P78213, MedChemExpress, duration: 48 h)), and pharmacological agonists or inhibitors (TMG (concentration #1–10 µm or concentration #2–15 µm, HY‐12588, MedChemExpress, duration: 48 h), OSMI (25 mm, HY‐119738, MedChemExpress, duration: 48 h), HY‐143404 (10 µm, MedChemExpress, duration: 48 h), ERK‐IN‐4 (50 µm, HY‐113592, MedChemExpress, duration: 48 h), SB‐431542 (10 µm, HY‐10431, MedChemExpress, duration: 48 h), CHX (50 µg mL^−1^, 66‐81‐9, Sigma–Aldrich, with time points as indicated in individual chase experiments), or MG132 (20 µm, HY‐13259, MedChemExpress, 6 h pretreatment). For gene silencing, cells were transfected with 100 pmol siRNA/shRNA using Lipofectamine 2000 for 24 h. Following transfection, cells were allowed to recover in complete medium for 48 h, and then started the above treatment. To facilitate a uniform description, the transfection time is hereafter collectively referred to as 72 h (24 h + 48 h). Stable transfectants were selected with 2 µg mL^−1^ puromycin. The sequences for (CBL, Tie2, USP20, GPR37, PSAP, and ACE‐specific shRNA were as followed: shACE, 5′‐TGGACACCCAGAAGGATATTT‐3′; shCBL, 5′‐GACAAGAAGATGGTGGAGAAG‐3′; shTie2 (GE Dharmacon, #V2LHS_93 160); shGPR37, 5′‐TCCAACGTCCAGATGTACTACCACACCTAGTACATCTGGACGTTGGTTTTTTC‐3′; shPSAP, 5′‐AAACTGTACGCCCTCGCCCTCTTC‐3′; shUSP20, 5′‐ CCGGCTATGTTGGCTGCGGAGAATCCTCGAGGATTCTCCGCAGCCAACATAGTTTTTG‐3′; shFbxw7, 5′‐CCAGAGAAATTGCTTGCTTTA‐3′; siSortilin, 5′‐ACCTCG CTGATAAGGATACAACAAGATCAAGAGTCTTGTTGTATCCTTATCAGCTT‐3′.

### Wb

Protein extracts were prepared using RIPA buffer (PC201plus, Epizyme Biomedical Technology, Shanghai, China) and quantified by BCA assay. After SDS‐PAGE separation and transfer to PVDF membranes, proteins were detected using specific primary antibodies (listed in Table S2, Supporting Information) followed by HRP‐conjugated secondary antibodies (AS011/AS007, Abclonal). Primary antibodies were incubated overnight at 4 °C, and secondary antibodies for 1 h at room temperature. Protein bands were visualized by ECL and quantified using ImageJ software.

### IF Staining

Cells grown on coverslips or tissue sections were fixed, permeabilized and blocked before incubation with primary antibodies overnight at 4 °C. After washing, samples were incubated with fluorescent secondary antibodies for 1 h at room temperature. Nuclei were counterstained with DAPI (C1006, Beyotime) and images were acquired using fluorescence microscopy.

### IHC

Deparaffinized sections were baked at 60 °C for 2 h, rehydrated through graded alcohols, and subjected to antigen retrieval in citrate buffer (pH 6.0, 0 05000, Thermo Fisher). Endogenous peroxidase was blocked with 3% H_2_O_2_. After serum blocking, sections were incubated with primary antibodies overnight at 4 °C, followed by secondary antibodies for 1 h at room temperature. DAB was used for color development and hematoxylin for counterstaining.

### NP‐RAW264.7 CoCulture

To comprehensively investigate NP cells‐macrophages interactions, both indirect (Transwell) and direct coculture systems were employed. For indirect coculture, shGPR37‐transfected RAW264.7 (2×10⁵ cells per well) were seeded in the upper chamber of 0.4 µm pore size Transwell inserts with complete DMEM/F12 medium (10% FBS, 1% penicillin‐streptomycin, 1% L‐glutamine), while NP cells (2×10⁵ cells/well) were cultured in the lower chamber, enabling soluble factor exchange without direct cell contact for 48 h under standard conditions (37 °C, 5% CO_2_). For direct coculture, RAW264.7 and NP cells were coseeded at 1:1 ratio (1×10⁵ cells each) on collagen‐coated chamber slides in 10%FBS DMEM/F12 medium, allowing direct cell–cell interactions mimicking native tissue microenvironments before 48‐h confocal imaging with antibodies against CD68 (for macrophages), PSAP, GPR37, and Shh (for NP cells). It should be declared that, because commercially available antibodies are subject to nonspecific‐staining limitations, the intrinsic cell morphology must be integrated with the fluorescence intensity of both intracellular and membrane markers for the two cell types to comprehensively identify macrophages and NP cells within the confocal microscope field.

### Co‐Immunoprecipitation (Co‐IP)

Cell lysates in IP buffer (P0013, Beyotime) were precleared with protein A/G agarose beads (Bio‐Rad) for 2 h at 4 °C. Lysates were then incubated with antibodies overnight at 4 °C, followed by protein A/G beads for 4 h. Precipitates were boiled in loading buffer and analyzed by Wb.

### Transcriptomics and O‐Glycoproteomic

The experimental design included six distinct sequencing comparisons: 1) Control versus ACE‐knockout rat NP cells to identify ACE‐regulated pathways and differentially expressed genes; 2) Control versus PSAP recombinant protein‐treated (10 µm) rat NP cells to characterize PSAP's direct effects; 3) Control versus PSAP recombinant protein‐treated (10 µm) RAW264.7 macrophages to examine intracellular signaling alterations; 4) Control versus PSAP recombinant protein‐treated (10 µm) RAW264.7 cells supernatants to analyze secretory changes, with particular focus on TGFβ expression; 5) Control versus TGFβ recombinant protein‐treated (10 ng mL^−1^) rat NP cells to investigate feedback mechanisms; and 6) Control versus TGFβ recombinant protein‐treated (10 ng mL^−1^) rat NP cells supernatants to analyze secretory changes, with particular focus on PSAP expression. All cellular transcriptomic analyses used RNA extracted from the respective cell types, while supernatant analysis focused on extracellular RNA profiles. For RNA isolation, NP cells samples were homogenized in TRIzol reagent (15 596 026, Invitrogen) followed by chloroform extraction and isopropanol precipitation. The RNA pellets were then purified using RNeasy Mini Kit columns (74 106, Qiagen) with on‐column DNase I treatment to eliminate genomic DNA contamination. RNA quality control was rigorously performed using Agilent Bioanalyzer 5400, with all samples meeting the stringent inclusion criteria of RNA Integrity Number (RIN) > 8.0, total mass > 0.2 ug. For library preparation, the Fast RNA‐seq Lib Prep Kit V2 (Cat. No. RK20306) was used following the manufacturer's protocol, with poly‐A selection for mRNA enrichment using VAHTS mRNA Capture Beads. The qualified libraries were sequenced on the Novaseq X Plus platform (150 bp paired‐end reads) by Novogene Co., Ltd. (Beijing, China), a professional sequencing service provider. Data were processed using the Limma package (|logFC|>0.5, p<0.05). Differentially expressed genes (DEGs) were analyzed by GO, KEGG, and GSEA using R software. O‐glycoproteomic were performed on shACE‐transfected and untreated rat‐derived NP cells. All identifiers of differentially expressed proteins (DEPs) and proteins corresponding to differentially expressed O‐glycosylation sites were systematically collected, with expression changes of O‐glycosylated proteins. Statistical significance was determined through Student's *t*‐test (*p* < 0.05) to identify DEPs between experimental and control groups. Subsequent functional characterization involved GO analysis categorizing DEPs into biological processes, cellular components, and molecular functions, while comprehensive functional annotation was performed using InterProScan software integrating Pfam, PRINTS, ProDom, SMART, ProSite, and PANTHER databases, complemented by COG and KEGG analyses for protein family classification and pathway mapping. Similarly, the O‐glycoproteomics analysis was also performed by Novogene Co., Ltd. as the service provider.

### IDD Animal Models

ACE‐knockout mice (C57BL/6J background, sourced from Jackson Laboratory) were interbred with wild‐type C57BL/6J mice (also from Jackson Laboratory) to produce ACE‐knockout and wild‐type control littermate animals. The experimental system utilized two viral vectors: 1) Lv‐OE‐PSAP (PSAP‐overexpressing lentivirus) and 2) AAV‐shGPR37‐F4/80‐EGFP (AAV vector expressing GPR37‐specific shRNA under control of the macrophage‐selective F4/80 promoter, enabling targeted GPR37 knockdown in macrophages while preserving its expression in other cell types), both at 1×10⁸ TU mL^−1^ (Genomeditech Co., Ltd.). The hydrogel materials were prepared using the previously reported the chitosan‐encapsulated virus hydrogel synthesis procedure.^[^ ^10^ ^]^ The composite hydrogel demonstrated good swelling capacity, biodegradability, biocompatibility, injectability, and controlled viral release capability. Three‐month‐old male SD rats (450±50 g, from Shanghai Shrek Experimental Animal Co., Ltd.) were randomly assigned according to the study protocol: control, IDD (needle puncture), hydrogel, Lv‐OE‐PSAP, and Lv‐OE‐PSAP+AAV‐sh‐GPR37‐F4/80‐EGFP groups (n = 6/group). After Co6/7‐disc puncture with 21G needle, treatments (2 µL hydrogel) were injected 1 week later. A ventilated environment with a photoperiod of 12:12 h and a constant temperature of 25 °C was provided for the experimental animals. After a predetermined period of time (4 and 8 weeks after intervention), a variety of IVD tissues were obtained from the rat tail during rat sacrifice. Imaging and histological examinations were conducted to determine the effect of the above treatments on IVD repair (4 and 8 weeks after intervention). This study received approval from Tongji University Ethics Committee (Approval No. TJBB05022101).

### Imaging and Histological Analysis

At 4 and 8 weeks post‐surgery, rat tails were scanned by X‐ray (Carestream) and 3.0T MRI (Philips). DHI% was calculated as: (D+E+F) × 100%/(A+B+C+D+E+F).^[^ ^82^ ^]^ A, B, and C are the heights of the anterior, midpoint, and posterior edges of the upper vertebral body, respectively. D, E, and F are the heights of the anterior, midpoint, and posterior edges of the intervertebral space, respectively. To assess disc rehydration, T2‐weighted MRI (3.0 T, GE Healthcare, USA) was performed at 4 weeks, and 8 weeks post‐intervention to evaluate implant efficacy. MRI acquisition parameters included: spin‐echo sequence with a repetition time of 3000 ms, echo time of 60 ms, 9 signal excitations, 1.5 mm slice thickness, and a 5 cm field of view. Disc rehydration was quantified using the MRI index, calculated as the product of NP area and signal intensity derived from midsagittal T2‐weighted images.^[^ ^83^ ^]^ For histology, IVDs were fixed in 4% PFA (G1107, Servicebio) for 48 h, decalcified using EDTA decalcification solution (10 009 617, Sinopharm) for 2 months, and stained with HE or SO (Solarbio). The degree of IVD was counted according to the grading scale as previously described.^[^ ^84^ ^]^

### Molecular Docking

AutoDock Vina 1.1.2 was employed to conduct molecular docking experiments with both the protein and the ligands that had already been modified (Trott and Olson, 2010). Docking analyses were conducted using PyMOL (The PyMOL Molecular Graphics System, version 4.40, Schrödinger LLC., New York, NY, USA) and discovery studio. The BIOVIA discovery studio visualizer (version 19, BIOVIA, San Diego, CA, USA) was employed to ascertain the molecules' binding energy and interaction.

### Subcellular Fractionation

Cells were collected and resuspended in a subcellular fractionation buffer composed of 250 mm sucrose, 10 mm KCl, 1.5 mm MgCl2, 20 mm HEPES (pH 7.4), 1 mm EDTA, 1 mm DTT, and 1 mm EGT, with the addition of a protease inhibitor cocktail. The cell suspension was initially centrifuged at 1,000 g for 5 min to obtain the postnuclear supernatant. This supernatant was subsequently centrifuged at 6,000 g for 5 min to isolate the mitochondrial fraction. The resultant supernatant from the 6,000 g centrifugation was further subjected to centrifugation at 20,000 g for 2 h to separate the membrane fraction, which was collected as the pellet. The supernatant remaining after the 20,000 g centrifugation was designated as the cytosolic fraction.

### Statistical Analysis

Statistical analyses were conducted utilizing GraphPad Prism (version 9.5.0), with data visualization performed using GraphPad Prism (version 9.5.0). The data presented in the graphs are expressed as mean ± standard deviation (SD). The statistical results were derived from a minimum of three biologically independent experimental replicates, with the sample size (n) specified in the figure legends. The Shapiro–Wilk test was employed to assess normality. Comparisons between two groups were made using the unpaired two‐tailed Student's *t*‐test, whereas comparisons among multiple groups were conducted using one‐way analysis of variance followed by Tukey's post hoc test. A p‐value of less than 0.05 was considered indicative of statistical significance. In the figures, asterisks denote significance levels as follows: ^*^ *p* < 0.05, ^**^ *p* < 0.01, ^***^ *p* < 0.001, while “ns” indicates a lack of statistical significance.

## Conflict of Interest

The authors declare no conflict of interest.

## Author Contributions

Y. G., F. W., B. W. contributed equally to this work. Y.G. and F.W. contributed to conceptualization. Y.G., F.W., and B.W. contributed to methodology. Y.G., F.W., and Y.Z. contributed to fabrication. Y.G., F.W., and C.W. contributed to investigation. Y.G. and F.W. prepared the original draft. T.H. and D.W. contributed to review and editing.

## Supporting information



Supporting Information

## Data Availability

The data that support the findings of this study are available from the corresponding author upon reasonable request.;
