# Supplementary material for: ACE‐mediated Glycosylation Stabilizes PSAP To Promote GPR37‐dependent Macrophage‐Nucleus Pulposus Cells Crosstalk and TGFβ Signaling in Alleviating Intervertebral Disc Degeneration
Source: Adv Sci (Weinh). 2025 Oct 14;12(43):e10662. doi: 10.1002/advs.202510662 (PMC12631909; doi:10.1002/advs.202510662)
Supplement: Supplementary file 1 — Supporting Information [file ADVS-12-e10662-s001.pdf]

Supporting Information

ACE-mediated Glycosylation Stabilizes PSAP To Promote GPR37-dependent Macrophage-Nucleus Pulposus Cells Crosstalk And TGFβ Signaling In Alleviating Intervertebral Disc Degeneration

Youfeng Guo, Feng Wang, Bijun Wang, Yu Zhou, Chao Wang, Tao Hu, Desheng Wu*

Table S1. Clinical information of human degenerative disc samples from 9 patients.

Variables	Patient 1	Patient 2	Patient 3	Patient 4	Patient 5	Patient 6	Patient 7	Patient 8	Patient 9
Sex	Female	Male	Male	Female	Male	Female	Male	Female	Male
Age	25	30	26	45	55	48	68	70	71
Diagnosis	Fracture	Fracture	Fracture	LDH	LDH	LSS	LSS	LSS	LSS
Level	L4/5	L5/S1	L5/S1	L4/5	L5/S1	L4/5	L5/S1	L5/S1	L4/5
Grades	II	II	II	III	III	III	V	V	V

LDH, lumbar disc degeneration; LSS, lumbar spinal stenosis.

Table S2. Antibodies used in this study

Antibody	Manufacturer	Catalog No.
COL2	Proteintech	28459-1-AP
p21	Proteintech	28248-1-AP
p16	Proteintech	10883-1-AP
ADAMTS4	Proteintech	11865-1-AP
ubiquitin	Proteintech	80992-1-RR
β-actin	Abclonal	AC026
Flag	Cell Signaling Technology	14793
Myc	Cell Signaling Technology	2276
HA	Cell Signaling Technology	3724
His	Cell Signaling Technology	12689
CBL	Abclonal	A22994
p-CBL	Abclonal	AP0794/ AP0780
ACE	Proteintech	24743-1-AP
Tie2	Proteintech	19157-1-AP
PI3K	Proteintech	20584-1-AP
p-PI3K	Abclonal	AP0854
AKT	Proteintech	10176-2-AP
p-AKT	Proteintech	66444-1-Ig
O-GlcNAc	Abcam	ab2739
OGT	Proteintech	11576-2-AP
ATP1A1	Proteintech	14418-1-AP
Sortilin	Proteintech	12369-1-AP
PSAP	Abcam	ab300469
α-tubulin	Proteintech	11224-1-AP

USP20	Proteintech	17491-1-AP
CD163	Proteintech	16646-1-AP
CD206	Proteintech	18704-1-AP
CD86	Proteintech	13395-1-AP
CD80	Proteintech	66406-1-Ig
ERK	Selleck	F0002
p-ERK	Selleck	A5036
MEK	Selleck	F0334
p-MEK	Selleck	F0345
TGFβ	Selleck	F1624
MMP2	Proteintech	10373-2-AP
MMP9	Proteintech	82854-8-RR
GPR37	Proteintech	14820-1-AP
ITGAV	Proteintech	27096-1-AP
ITGB6	Proteintech	28378-1-AP
Smad2	Selleck	F0309
pSmad2	Selleck	F1689
Smad3	Selleck	F0364
pSmad3	Abclonal	AP0727
Fbxw7	Abclonal	A5872
Shh	Abcam	ab308225

COL2, Collagen type II; ADAMTS4, A disintegrin and metalloproteinase with thrombospondin motifs 4; CBL, Casitas B-lineage lymphoma proto-oncogene; ACE, Angiotensin-converting enzyme; Tie2, TEK receptor tyrosine kinase; PI3K, Phosphatidylinositol 3-kinase; Akt, Protein kinase B; OGT, O-linked N-acetylglucosamine transferase; ATP1A1, ATPase Na+/K+ transporting subunit alpha 1; Sortilin, Sortilin 1; PSAP, Prosaposin; α-tubulin, Alpha tubulin; USP20, Ubiquitin specific peptidase 20; ERK, Extracellular signal-regulated kinase; MEK, Mitogen-activated protein kinase; TGFβ, Transforming growth factor beta; MMP2, Matrix metalloproteinase 2; MMP9, Matrix metalloproteinase 9; GPR37, G protein-coupled receptor 37; ITGAV, Integrin subunit alpha V; ITGB6, Integrin subunit beta 6; Fbxw7, F-box and WD repeat domain-containing 7; Shh, sonic hedgehog signaling molecule .

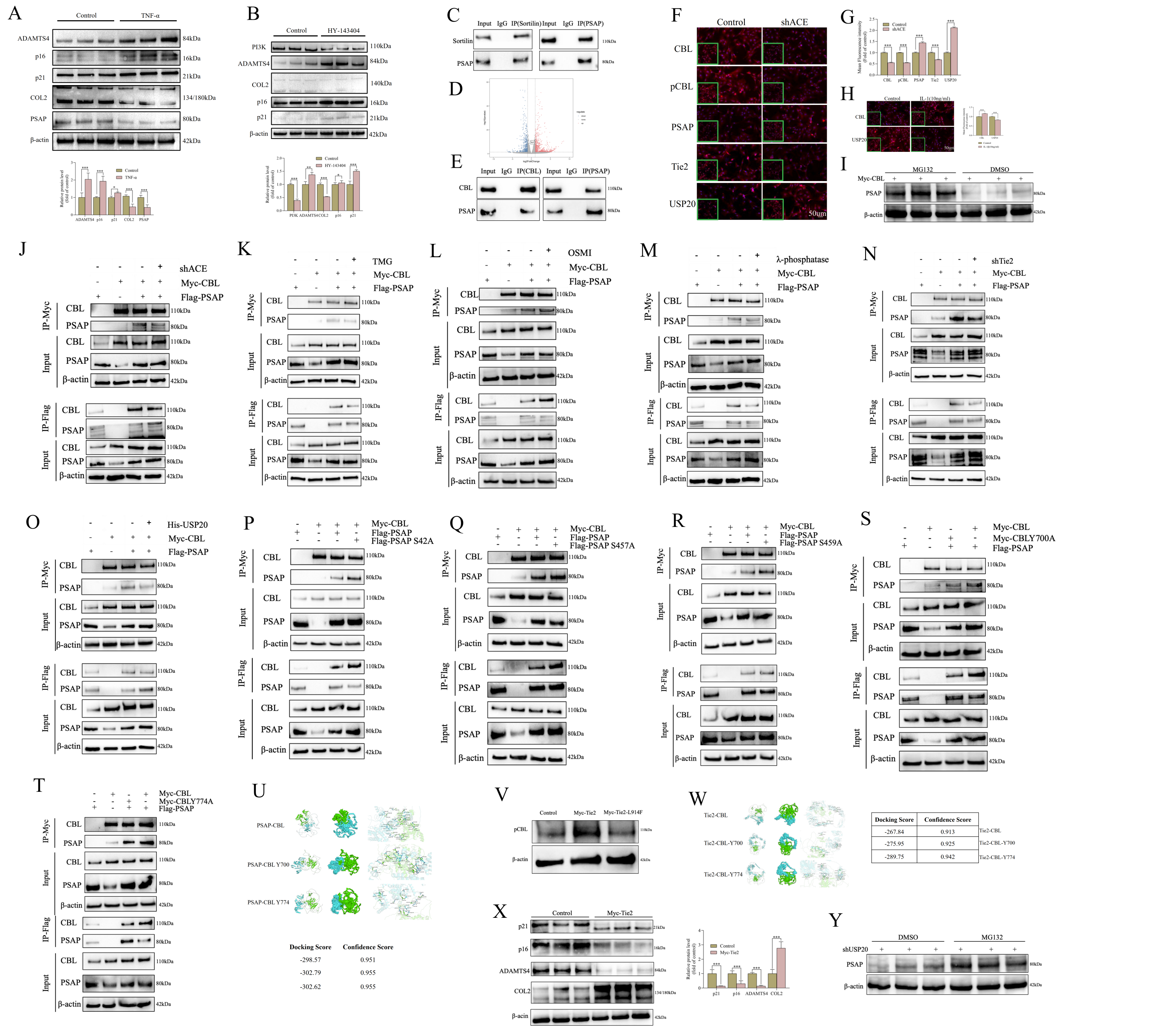

Figure S1. Changes in the interaction between CBL and PSAP under different intervention conditions. A: After TNF-α (10 ng/mL) treatment of SD rat-derived NP cells in vitro, protein levels of PSAP, degeneration markers (COL2 and ADAMTS4) and senescence markers (p16 and p21) in NP cells were detected. Band gray values were semi-quantitatively analyzed using ImageJ (n=3). B: After PI3K inhibitor HY-143404 (10 μM) treatment of SD rat-derived NP cells in vitro, protein levels of PSAP, degeneration markers (COL2 and ADAMTS4) and senescence markers (p16 and p21) in NP cells were detected. Band gray values were semi-quantitatively analyzed using ImageJ (n=3). C: Co-IP validation of Sortilin and PSAP. D: Transcriptome analysis of Sortilin differential expression after shACE (lentivirus carrying ACE shRNA) transfection of SD rat-derived NP cells in vitro. E: Co-IP validation of CBL and PSAP. F-G: After shACE transfection of SD rat-derived NP cells in vitro, cellular IF staining was used to detect total CBL protein and its phosphorylated protein, PSAP and USP20 protein levels. Fluorescence intensity was semi-quantitatively analyzed using ImageJ (n=3). H: After IL-1β (10 ng/mL) treatment of SD rat-derived NP cells in vitro, cellular IF staining was used to detect CBL and USP20 protein levels. Fluorescence intensity was semi-quantitatively analyzed using ImageJ (n=3). I: SD rat-derived NP cells were treated with Myc-tagged CBL overexpression plasmid (Myc-CBL) for 72 hours, followed by treatment with MG 132 (20 μM) for 6 hours. J: Myc-CBL, Flag-tagged PSAP overexpression plasmid (Flag-PSAP) and shACE were co-transfected into SD rat-derived NP cells, followed by IP with Myc or Flag antibody, then IB with PSAP and CBL antibodies. K: Myc-CBL and Flag-PSAP were co-transfected into SD rat-derived NP cells and treated with TMG (10 μM), followed by IP with Myc or Flag antibody, then IB with PSAP and CBL antibodies. L: Myc-CBL and Flag-PSAP were co-transfected into SD rat-derived NP cells and treated with OSMI (25 mM), followed by IP with Myc or Flag antibody, then IB with PSAP and CBL antibodies. M: Myc-CBL and Flag-PSAP were co-transfected into SD rat-derived NP cells and treated with phosphatase, followed by IP with Myc or Flag antibody, then IB with PSAP and CBL antibodies. N: Myc-CBL, Flag-PSAP and shTie2 (lentivirus carrying Tie2 shRNA) were co-transfected into SD rat-derived NP cells, followed by IP with Myc or Flag antibody, then IB with PSAP and CBL antibodies. O: Myc-CBL, Flag-PSAP and His-tagged USP20 overexpression plasmid (His-USP20) were co-transfected into SD rat-derived NP cells, followed by IP with Myc or Flag antibody, then IB with PSAP and CBL antibodies. P: Myc-CBL, Flag-PSAP and Flag-tagged PSAP overexpression mutant plasmid (Flag-PSAP S42A) were co-transfected into SD rat-derived NP cells, followed by IP with Myc or Flag antibody, then IB with PSAP and CBL antibodies. Q: Myc-CBL, Flag-PSAP and Flag-PSAP S457A were co-transfected into SD rat-derived NP cells, followed by IP with Myc or Flag antibody, then IB with PSAP and CBL antibodies. R: Myc-CBL, Flag-PSAP and Flag-PSAP S459A were co-transfected into SD rat-derived NP cells, followed by IP with Myc or Flag antibody, then IB with PSAP and CBL antibodies. S: Myc-CBL, Flag-PSAP and Myc -tagged CBL overexpression plasmid (Myc-CBL Y700A) were co-transfected into SD rat-derived NP cells, followed by IP with Myc or Flag antibody, then IB with PSAP and CBL antibodies. T: Myc-CBL, Flag-PSAP and Myc-CBL Y774A were co-transfected into SD rat-derived NP cells, followed by IP with Myc or Flag antibody, then IB with PSAP and CBL antibodies. U: Molecular docking of dynamic modification omics was used to detect the binding free energy between phosphorylated CBL and PSAP. The green structure represents the CBL protein, and the blue structure represents the PSAP protein. V: After transfection of SD rat-derived NP cells with Myc-tagged Tie2 overexpression plasmid (Myc-Tie2) or Myc-tagged Tie2 overexpression kinase-dead mutant plasmid (Myc-Tie2 L914F), pCBL was detected. W: Molecular docking of dynamic modification omics was used to detect the binding free energy between phosphorylated CBL and Tie2. The green structure represents the CBL protein, while the blue structure corresponds to the Tie2 protein. X: After Myc-Tie2 transfection of SD rat-derived NP cells in vitro, protein levels of degeneration (COL2 and ADAMTS4) and senescence (p16 and p21) phenotype markers in NP cells were detected. Band gray values were semi-quantitatively analyzed using ImageJ (n=3). Y: SD rat-derived NP cells were treated with shUSP20 (lentivirus carrying USP20 shRNA) for 72 hours, followed by treatment with MG 132 (20 μM) for 6 hours. All data are expressed as the mean ± SD. Comparisons between two groups were made using the unpaired two-tailed Student's t-test, whereas comparisons among multiple groups were conducted using one-way analysis of variance followed by Tukey's post hoc test. A p-value of less than 0.05 was considered indicative of statistical significance. *$p < 0.05$, **$p < 0.01$, ***$p < 0.001$, while "ns" indicates a lack of statistical significance. ACE, Angiotensin-converting enzyme; PSAP, Prosaposin; Tie2, TEK receptor tyrosine kinase; CBL, Casitas B-lineage lymphoma; USP20, Ubiquitin-specific-processing protease 20; NP, Nucleus pulposus; COL2, Collagen type II; ADAMTS4, A Disintegrin and Metalloproteinase with Thrombospondin Motifs 4; p16, Cyclin-dependent kinase inhibitor 2A; p21, Cyclin-dependent kinase inhibitor 1; MG 132, Carbobenzoxy-Leu-Leu-leucinal; Co-IP, Co-immunoprecipitation; IF, Immunofluorescence; TMG, Thiamet-G; OSMI, O-Glycosyltransferase inhibitor; IP, Immunoprecipitation; IB, Immunoblotting.

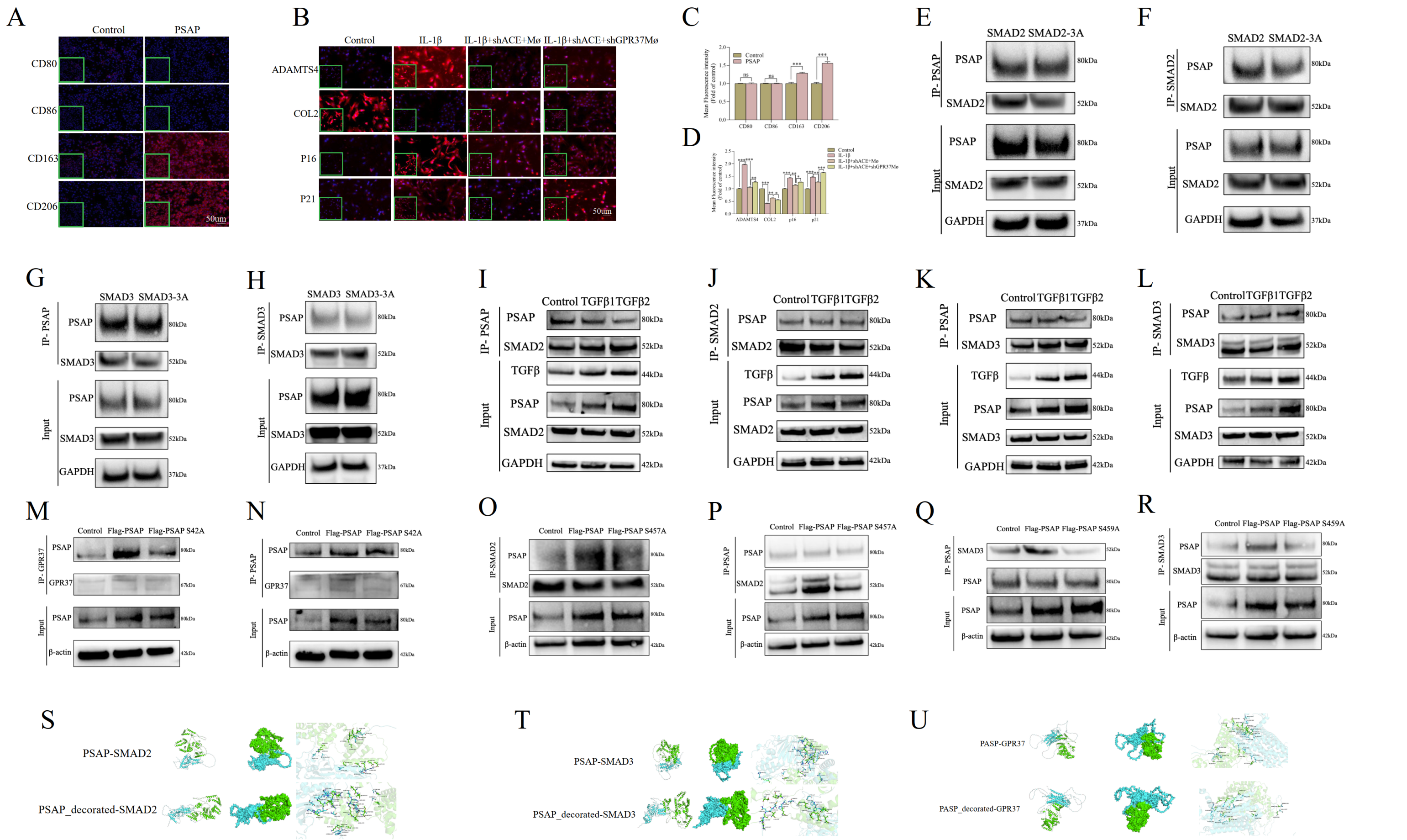

	Docking Score	Confidence Score
PSAP-SMAD2	-257.85	0.896
PSAP_decorated-SMAD2	-397.09	0.993

	Docking Score	Confidence Score
PSAP-SMAD3	-279.92	0.931
PSAP_decorated-SMAD3	-377.9	0.990

Docking Score	Confidence Score	
-294.95	0.948	PASP-GPR37
-412.52	0.995	PASP_decorated-GPR37

Figure S2. Regulation of macrophage polarization by PSAP and its mutual regulation with TGFβ/SMAD2-3. A and C: After PSAP recombinant protein (10 μM) treatment of RAW264.7 cells in vitro, cellular IF staining was used to detect M1 and M2 type macrophage marker protein levels. Fluorescence intensity was semi-quantitatively analyzed using ImageJ (n=3). B and D: After IL-1β (10 ng/mL) modeling of SD rat-derived NP cells in vitro, shACE (lentivirus carrying ACE shRNA) transfection was performed, followed by co-culture with RAW264.7 or RAW264.7 transfected with shGPR37 (lentivirus carrying GPR37 shRNA), then cellular IF staining was used to detect protein levels of degeneration and senescence phenotype markers in SD rat-derived NP cells. Fluorescence intensity was semi-quantitatively analyzed using ImageJ (n=3). E-F: SMAD2 WT or SMAD2-3A was transfected into RAW264.7 cells, followed by IP with PSAP or SMAD2 antibody, then IB with SMAD2 and PSAP antibodies. G-H: SMAD3 WT or SMAD3-3A was transfected into RAW264.7 cells, followed by IP with PSAP or SMAD3 antibody, then IB with SMAD3 and PSAP antibodies. I-J: RAW264.7 cells treated with different concentration gradients of TGFβ (concentration #1: 5 ng/mL or concentration #2: 10 ng/mL) were subjected to IP with PSAP or SMAD2 antibody, then IB with SMAD2 and PSAP antibodies. K-L: RAW264.7 cells treated with different concentration gradients of TGFβ (concentration #1: 5 ng/mL or concentration #2: 10 ng/mL) were subjected to IP with PSAP or SMAD3 antibody, then IB with SMAD3 and PSAP antibodies. M-N: Flag-tagged PSAP overexpression plasmid (Flag-PSAP) or Flag-tagged PSAP overexpression mutant plasmid (Flag-PSAP S42A) was transfected into RAW264.7 cells, followed by IP with PSAP or GPR37 antibody, then IB with PSAP and GPR37 antibodies. O-P: Flag-PSAP or Flag-PSAP S457A was transfected into RAW264.7 cells, followed by IP with PSAP or SMAD2 antibody, then IB with PSAP and SMAD2 antibodies. Q-R: Flag-PSAP or Flag-PSAP S459A was transfected into RAW264.7 cells, followed by IP with PSAP or SMAD3 antibody, then IB with PSAP and SMAD3 antibodies. S: Molecular docking of dynamic modification omics was used to detect the binding free energy between glycosylated PSAP and SMAD2. The green structure represents the PSAP protein, while the blue structure corresponds to the SMAD2 protein. T: Molecular docking of dynamic modification omics was used to detect the binding free energy between glycosylated PSAP and SMAD3. The green structure represents the PSAP protein, while the blue structure corresponds to the SMAD3 protein. U: Molecular docking of dynamic modification omics was used to detect the binding free energy between glycosylated PSAP and GPR37. The green structure represents the PSAP protein, while the blue structure corresponds to the GPR37 protein. All data are expressed as the mean ± SD. Comparisons between two groups were made using the unpaired two-tailed Student's t-test, whereas comparisons among multiple groups were conducted using one-way analysis of variance followed by Tukey's post hoc test. A p-value of less than 0.05 was considered indicative of statistical significance. $*p < 0.05$, $**p < 0.01$, $***p < 0.001$, while "ns" indicates a lack of statistical significance. PSAP, Prosaposin; TGFβ, Transforming growth factor β; SMAD2, Mothers against decapentaplegic homolog 2; SMAD3, Mothers against decapentaplegic homolog 3; IF, Immunofluorescence; NP, Nucleus pulposus; IL-1β, Interleukin-1β; GPR37, G protein-coupled receptor 37; IP, Immunoprecipitation; IB, Immunoblotting; ACE, Angiotensin-converting enzyme.

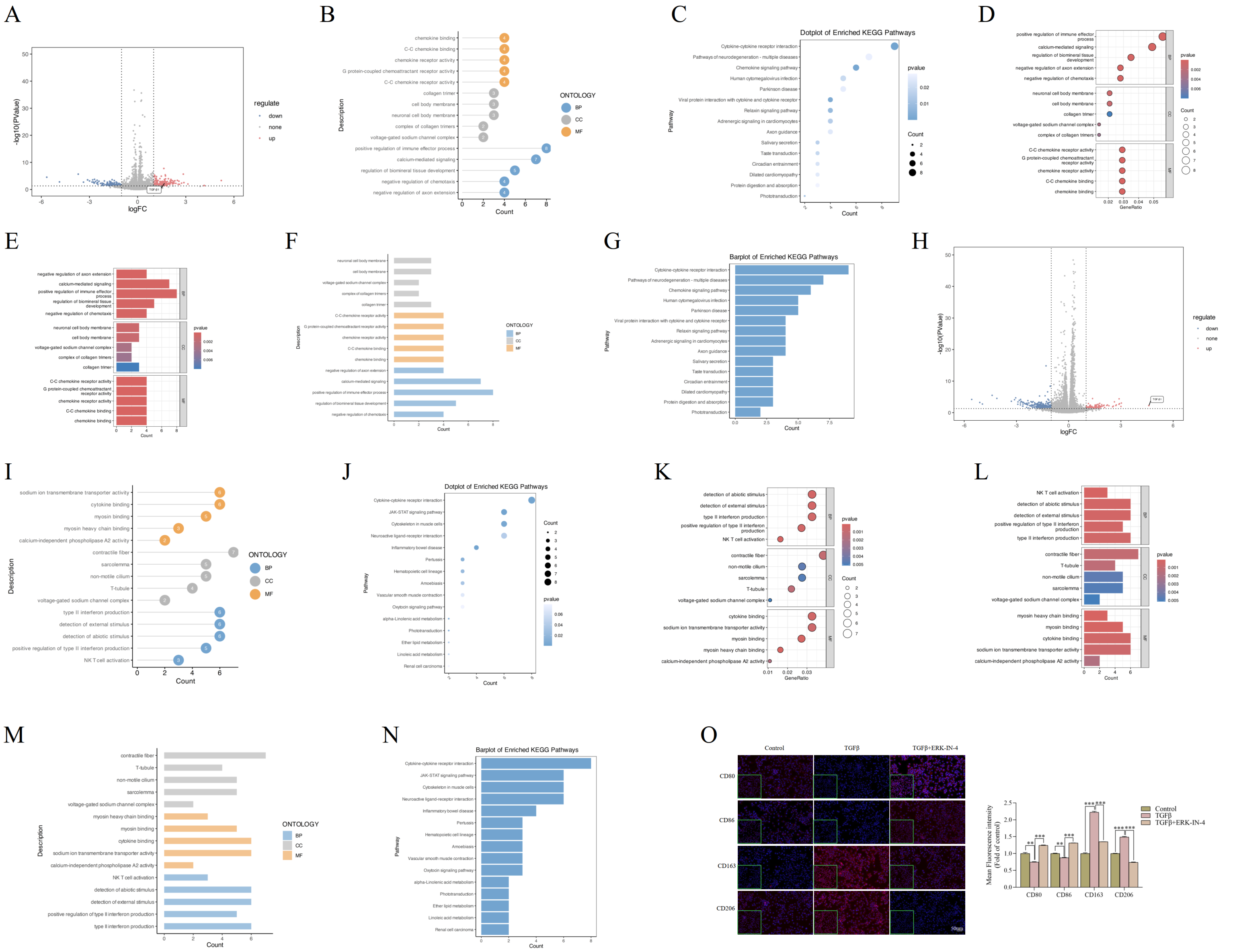

Figure S3. Transcriptome analysis functional verification of PSAP-regulated macrophages. A-G: After PSAP recombinant protein (10 μM) treatment of RAW264.7 cells in vitro, transcriptome sequencing was performed to analyze differential genes (A), GO (B, D, E, and F) and KEGG (C and G) functional pathway enrichment, etc. H-N: After PSAP recombinant protein (10 μM) treatment of RAW264.7 cells in vitro, transcriptome sequencing of the supernatant was performed to analyze differential genes (H), GO (I, K, L, and M) and KEGG (J and N) functional pathway enrichment, etc. O: After treatment of RAW264.7 cells with TGFβ recombinant protein (10 ng/mL) or TGFβ recombinant protein (10 ng/mL) combined with ERK inhibitor ERK-IN-4 (50 μM) in vitro, cellular IF staining was used to detect M1 and M2 type macrophage marker protein levels. Fluorescence intensity was semi-quantitatively analyzed using ImageJ (n=3). All data are expressed as mean ± SD. Comparisons between two groups were made using the unpaired two-tailed Student's t-test, whereas comparisons among multiple groups were conducted using one-way analysis of variance followed by Tukey's post hoc test. A p-value of less than 0.05 was considered indicative of statistical significance. *p < 0.05, **p < 0.01, ***p < 0.001, while "ns" indicates a lack of statistical significance. PSAP, Prosaposin; KEGG, Kyoto Encyclopedia of Genes and Genomes; GO, Gene Ontology; ERK, Extracellular signal-regulated kinase; TGFβ, Transforming growth factor β; IF, Immunofluorescence.

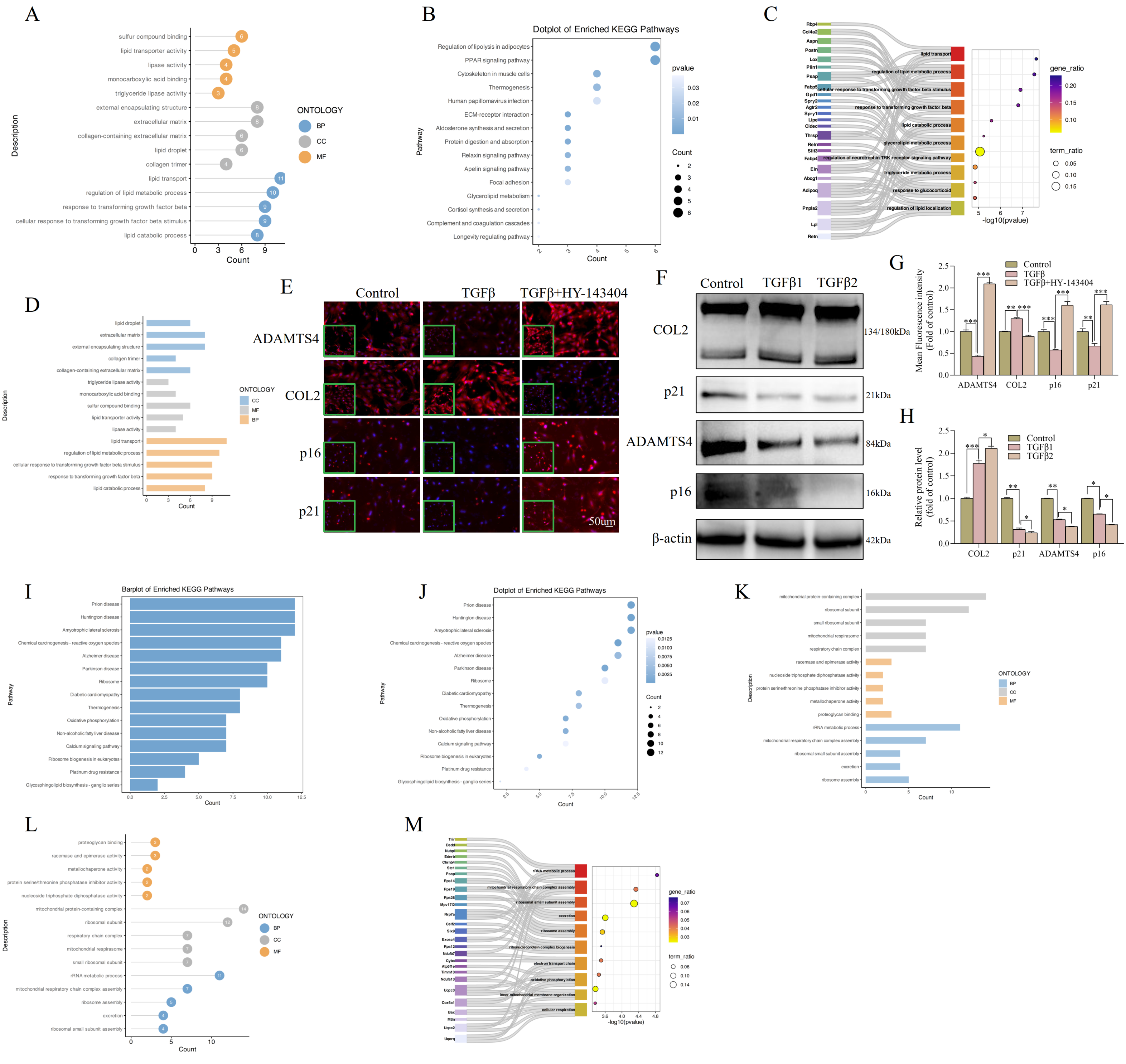

Figure S4. Regulatory mechanism of TGFβ on NP cells. A-D: After TGFβ recombinant protein (10 ng/mL) treatment of SD rat-derived NP cells in vitro, transcriptome sequencing was performed to analyze differential genes, GO (A and D) and KEGG (B and C) functional pathway enrichment, etc. E and G: After treatment of SD rat-derived NP cells with TGFβ recombinant protein (10 ng/mL) or TGFβ recombinant protein (10 ng/mL) combined with PI3K inhibitor HY-143404 (10 μM) in vitro, cellular IF staining was used to detect protein levels of degeneration (COL2 and ADAMTS4) and senescence (p16 and p21) markers. Fluorescence intensity was semi-quantitatively analyzed using ImageJ (n=3). F and H: After treatment of SD rat-derived NP cells with different concentration gradients of TGFβ recombinant protein (concentration #1: 5 ng/mL or concentration #2: 10 ng/mL) in vitro, Wb was used to detect protein levels of degeneration (COL2 and ADAMTS4) and senescence (p16 and p21) markers. Band gray values were semi-quantitatively analyzed using ImageJ (n=3). I-M: After TGFβ recombinant protein (10 ng/mL) treatment of SD rat-derived NP cells in vitro, transcriptome sequencing of the supernatant was performed to analyze differential genes, KEGG (I and J) and GO (K, L, and M) functional pathway enrichment, etc. All data are expressed as mean ± SD. Comparisons between two groups were made using the unpaired two-tailed Student's t-test, whereas comparisons among multiple groups were conducted using one-way analysis of variance followed by Tukey's post hoc test. A p-value of less than 0.05 was considered indicative of statistical significance. *p < 0.05, **p < 0.01, ***p < 0.001, while "ns" indicates a lack of statistical significance. NP, Nucleus pulposus; IF, Immunofluorescence; TGFβ, transforming growth factor β; PI3K, Phosphoinositide 3-Kinase; KEGG, Kyoto Encyclopedia of Genes and Genomes; GO, Gene Ontology; COL2, Collagen type II; ADAMTS4, A Disintegrin and Metalloproteinase with Thrombospondin Motifs 4; p16, Cyclin-dependent kinase inhibitor 2A; p21, Cyclin-dependent kinase inhibitor 1.

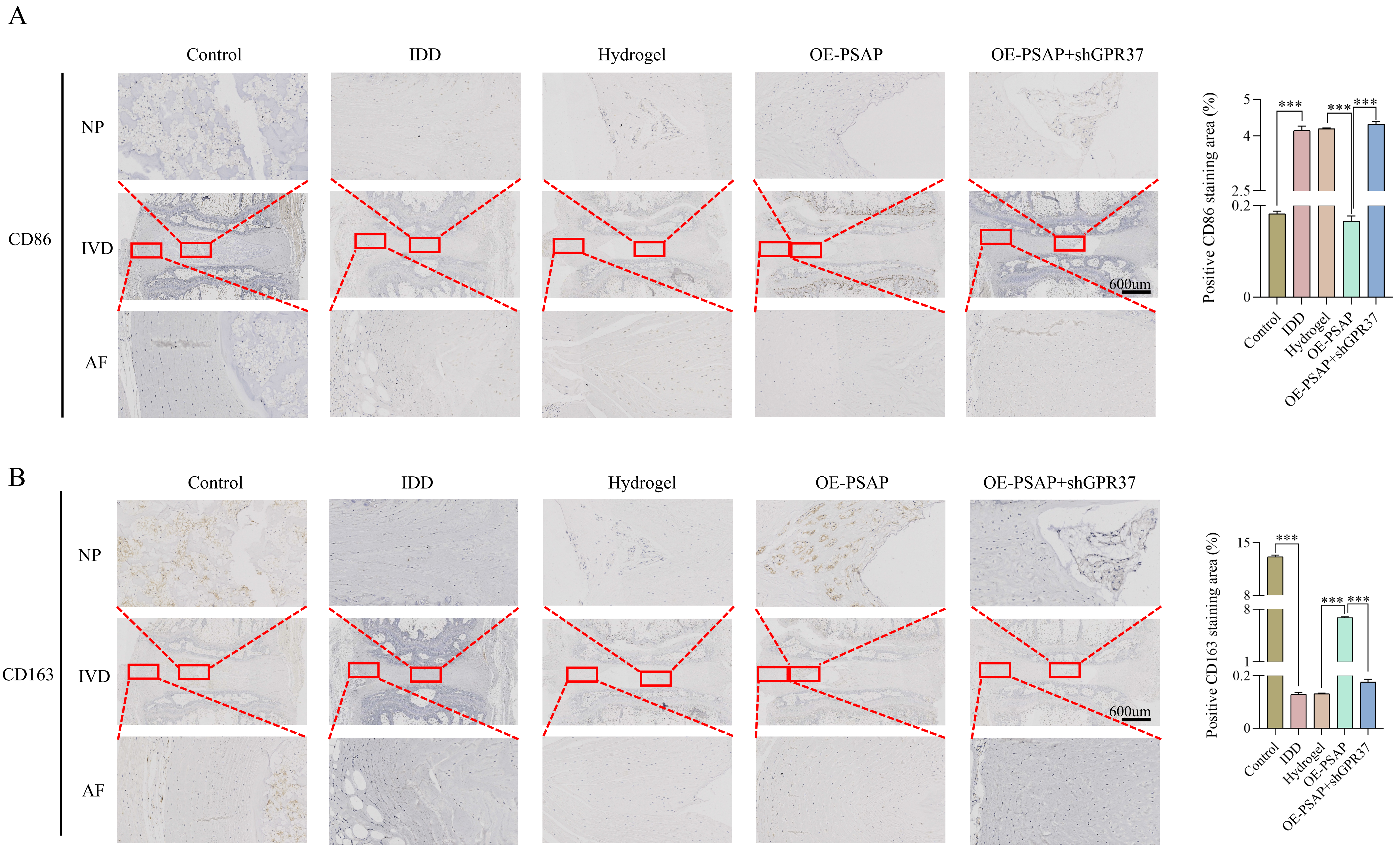

Figure S5. Effects of virus-loaded composite hydrogel on macrophage infiltration of rat tailbone IVD corresponding to 8 weeks after intervention. A-B: Macrophage infiltration (M1-CD86 and M2-CD163) IHC staining detection and quantitative analysis of IHC staining at 8 weeks after intervention (n=6). All data are expressed as mean ± SD. Comparisons between two groups were made using the unpaired two-tailed Student's t-test, whereas comparisons among multiple groups were conducted using one-way analysis of variance followed by Tukey's post hoc test. A p-value of less than 0.05 was considered indicative of statistical significance. *p < 0.05, **p < 0.01, ***p < 0.001, while "ns" indicates a lack of statistical significance. IHC, Immunohistochemistry; CD86, Cluster of Differentiation 86; CD163, Cluster of Differentiation 163; IVD, Intervertebral disc.
